# Differential impact of non-pharmaceutical public health interventions on COVID-19 epidemics in the United States

**DOI:** 10.1186/s12889-021-10950-2

**Published:** 2021-05-21

**Authors:** Xiaoshuang Liu, Xiao Xu, Guanqiao Li, Xian Xu, Yuyao Sun, Fei Wang, Xuanling Shi, Xiang Li, Guotong Xie, Linqi Zhang

**Affiliations:** 1 Ping An Healthcare Technology, Beijing, China; 2 Center for Global Health and Infectious Diseases, School of Medicine, Tsinghua University, Beijing, China; 3 Department of Healthcare Policy and Research, Weill Cornell Medicine, Cornell University, New York, USA; 4 Ping An Health Cloud Company Limited, Beijing, China; 5 Ping An International Smart City Technology Co., Ltd., Beijing, China

**Keywords:** COVID-19, Non-pharmaceutical public health interventions, Reproduction number, Epidemic control, The United States

## Abstract

**Background:**

The widespread pandemic of novel coronavirus disease 2019 (COVID-19) poses an unprecedented global health crisis. In the United States (US), different state governments have adopted various combinations of non-pharmaceutical public health interventions (NPIs), such as non-essential business closures and gathering bans, to mitigate the epidemic from February to April, 2020. Quantitative assessment on the effectiveness of NPIs is greatly needed to assist in guiding individualized decision making for adjustment of interventions in the US and around the world. However, the impacts of these approaches remain uncertain.

**Methods:**

Based on the reported cases, the effective reproduction number (*R*_*t*_) of COVID-19 epidemic for 50 states in the US was estimated. Measurements on the effectiveness of nine different NPIs were conducted by assessing risk ratios (RRs) between *R*_*t*_ and NPIs through a generalized linear model (GLM).

**Results:**

Different NPIs were found to have led to different levels of reduction in *R*_*t*_. Stay-at-home contributed approximately 51% (95% CI 46–57%), wearing (face) masks 29% (15–42%), gathering ban (more than 10 people) 19% (14–24%), non-essential business closure 16% (10–21%), declaration of emergency 13% (8–17%), interstate travel restriction 11% (5–16%), school closure 10% (7–14%), initial business closure 10% (6–14%), and gathering ban (more than 50 people) 7% (2–11%).

**Conclusions:**

This retrospective assessment of NPIs on *R*_*t*_ has shown that NPIs played critical roles on epidemic control in the US in the past several months. The quantitative results could guide individualized decision making for future adjustment of NPIs in the US and other countries for COVID-19 and other similar infectious diseases.

**Supplementary Information:**

The online version contains supplementary material available at 10.1186/s12889-021-10950-2.

## Background

Coronavirus disease 2019 (COVID-19), caused by the severe acute respiratory syndrome coronavirus 2 (SARS-COV-2), has become a global pandemic. Currently, the epidemic in the United States (US) is still of serious concern [1]. As of June 27, 2020, there have been 2,596,537 reported cases in the US, with 128,152 deaths [2]. The daily number of reported new cases has recently been rising again after a period of decline. Between February and April, 2020, various non-pharmaceutical public health interventions (NPIs) were adopted in different US states. However, they were occasionally challenged by local governments and the public due to high economic and lifestyle costs [3–5]. Notably, since April 20, 2020, all 50 states have been gradually relaxing NPIs. However, due to the lack of data supporting the actual effect of each NPIs, unsuitable policy relaxation may cause an even more serious pandemic. For example, a substantial increase in daily new cases was observed in Texas after the relaxation of stay-at-home on April 30th. Therefore, a retrospective quantitative assessment of the impacts of individual interventions on epidemic control is of great importance. This could assist policymakers and health care providers in making informed decisions on future adjustment of NPIs for COVID-19 and other infectious diseases transmitted via similar routes.

This study leveraged the combined NPIs being executed in different states to estimate the effects of individual NPIs implemented for epidemic containment among US states, including declaration of emergency, school closure, gathering ban (more than 10 or 50 people), initial business closure (e.g., dine-in service and retail), non-essential business closure, interstate travel restriction, wearing face masks, and stay-at-home orders.

## Methods

**Fig. 1:**
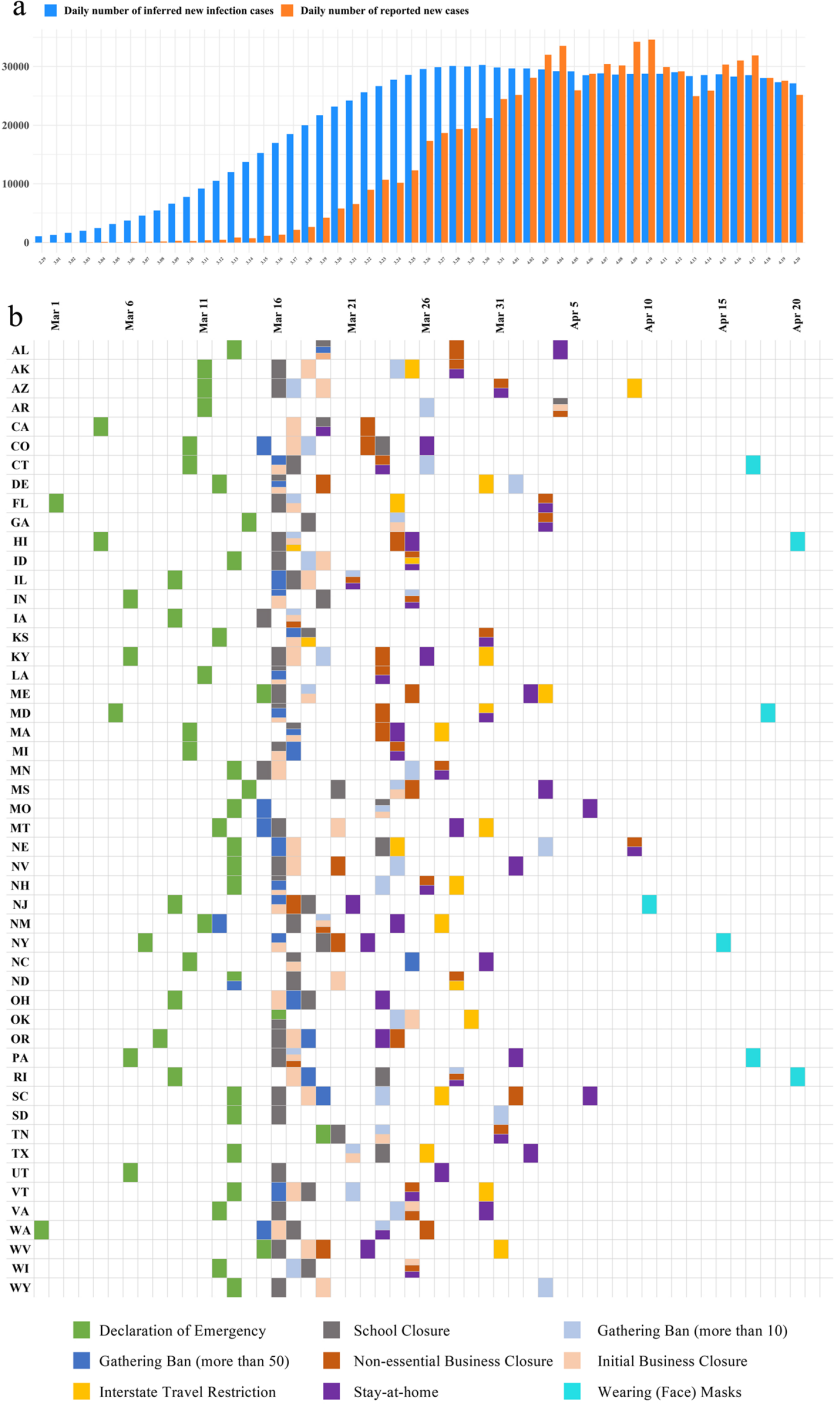
Daily number of reported and inferred new cases in the US and execution dates of main NPIs among 50 states. **a**. Daily number of reported new cases and inferred new infection cases (up to April 20) in the US (50 states). **b**. Execution dates of the selected main NPIs among 50 states in the US from February 29 to April 20. Interventions that started on the same day are represented in one rectangle. The full names of the state abbreviations are shown in Supplemental table 1

The number of reported cases in the US (50 states) from January 21 to May 31, 2020 was collected for this study [6]. Considering the time delay between infection time and reporting time, including an incubation period (assumed to follow a gamma distribution with mean = 5.1 days and SD = 3.0 days [7]) and a reporting delay (assumed to follow a gamma distribution with mean = 4.9 days and SD = 3.3 days [8]), the infection epidemic curves were inferred by randomly selecting samples from the two gamma distributions. The total daily number of reported new cases and inferred new infection cases in all 50 states during this period are shown in Fig. 1a, along with the timeline of Federal NPI responses. Due to the fact that states have been gradually relaxing interventions since April 20, 2020, the inferred infection numbers for each state prior to April 20th were used for final estimates of the daily effective reproduction number (*R*_*t*_). Relationships among these time-related concepts are shown in Supplemental fig. 1a. The method proposed by Thompson R et al. [9] was applied to estimate the time varying *R*_*t*_ for each state (R software package EpiEstim) from the inferred infection epidemic curves over seven-day sliding windows. The generation time used for the calculation of *R*_*t*_ was obtained from the time lag between the 133 collected infector/infectee pairs through a gamma distribution using maximum likelihood estimation (mean: 5.9, SD: 3.9, Supplemental fig. 1b, Supplemental table 3, R software package R0). To examine the association between *R*_*t*_ and NPIs, the timeline of the most widely executed NPIs for each state from February 29th to April 20th were collected (Fig. 1b, Supplemental table 1, 2). Note that inclusion relations exist for certain selected interventions, meaning that one intervention would be implemented by default if another more aggressive intervention was issued. Here, three such relations were identified: 1) gathering ban (more than 50 people) is included in gathering ban (more than 10 people); 2) initial business closure is included in non-essential business closure; 3) stay-at-home covers all other interventions except for declaration of emergency, interstate travel restriction and wearing (face) masks. The generalized linear model (GLM) with ridge regression for the gamma distribution (glmGamma) was developed to estimate the impacts of different interventions on *R*_*t*_ (R package H2o). For each state, the population density (number of people per square mile) [10], per capita GDP [11], median age [12], testing rate, and testing positive rate [6] were considered potential confounders. The risk ratios (RRs) of different interventions were then calculated from the coefficients, representing the impact of interventions on *R*_*t*_.

## Results

**Fig. 2:**
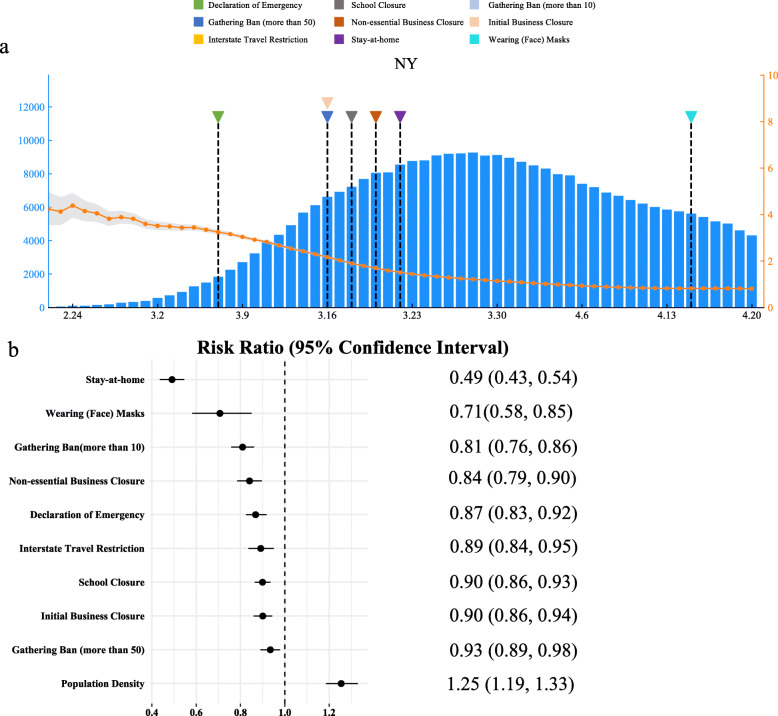
Time varying *R*_*t*_ and inferred infection epidemic curve for New York state (**a**) and risk ratios of each variable to *R*_*t*_ (**b**). **a**. Time varying *R*_*t*_ and inferred infection epidemic curve of New York state (NY). The blue bars represent the daily number of infections, the orange lines show the trends of *R*_*t*_ (standard deviation less than 0.5), and the grey shading refers to the 95% confidence intervals of *R*_*t*_. The dates of the main NPIs executed by NY from February 22 (1 week before the first state emergency on February 29) to April 20 were shown in different colors of triangles. **b**. Risk ratios (dot) and 95% confidence intervals (bars) of each variable to *R*_*t*_

The infection epidemic curves were first estimated for all 50 states, with consideration of the incubation period and reporting delays, based on the number of daily reported new cases from January 21 to May 31, 2020. The *R*_*t*_ of each state was calculated accordingly, from the first inferred case to April 20th (exemplified by New York (NY) State in Fig. 2a; others in Supplemental fig. 2). Some states started with high *R*_*t*_ values in the early phase that then fell below 1.0 on April 20th (Supplemental fig. 3) after implementation of aggressive NPIs. Taking NY as an example, *R*_*t*_ decreased from 3.25 (95% CI 3.17–3.32) on March 7th (declaration of emergency) to 1.52 (1.50–1.53) on March 22nd (stay-at-home), then fell below 1.0 on April 4th.

When constructing the GLM model for effect estimation, only population density was statistically significant (*p* < 0.0001), while *P*-values of the other pre-defined confounders were above 0.1 (per capita GDP 0.45; median age, 0.20; testing rate, 0.87; testing positive rate, 0.35). In the final model, only population density was selected as a confounder. Based on the constructed GLM model (R-squared = 0.79), risk ratios (RRs) of each NPI with respect to *R*_*t*_ were assessed (Fig. 2b). Due to the inclusion relation of some interventions, the actual RR of a more aggressive intervention was recalculated by accumulating the multivariable adjusted RRs of the included interventions (Supplemental table 4). Based on the constructed GLM model, stay-at-home exhibited the lowest RR value of 0.49 (95% CI 0.43–0.54), indicating that the execution of this NPI could reduce the current *R*_*t*_ by about 51% (46–57%). The reduction in *R*_*t*_ generated by wearing (face) masks, gathering ban (more than 10 people), non-essential business closure, and declaration of emergency were 29% (15–42%), 19% (14–24%), 16% (10–21%), and 13% (8–17%), respectively. Interstate travel restriction, school closure, and initial business closure achieved reductions in *R*_*t*_ corresponding to 11% (5–16%), 10% (7–14%) and 10% (6–14%), respectively. Gathering ban (more than 50 people) had the smallest effect (*R*_*t*_ (7, 2–11%) among the nine interventions.

## Discussion

This study quantitatively estimated the impacts of different NPIs on *R*_*t*_ in the US and ranked nine selected interventions in terms of their potential capacities to decrease *R*_*t*_ and control the COVID-19 epidemic. We have demonstrated that the selected nine interventions substantially reduce *R*_*t*_, which represents control of a COVID-19 outbreak. The analysis in this study reveals which interventions were more effective in controlling epidemics in the US. Stay-at-home produced the greatest reduction in *R*_*t*_ of about 51% (95% CI 46–57%). This covers a series of strong controls for maintaining social distancing, including, but not limited to, restricting gathering sizes to 10 or 50 people and closing initial businesses, non-essential businesses, and schools. As a basic NPI measure, wearing (face) masks was found to reduce *R*_*t*_ by about 29% (15–42%). Some studies have also found that wearing a face mask can be effectively combined with social distancing to flatten the epidemic curve [13]. By integrating 12,710 samples from more than 50 countries in the world, Barasheed et al. found that wearing masks in crowded places could reduce the risk of respiratory infections by 20% [14]. Interstate travel restriction and closing schools were found to moderately reduce *R*_*t*_ by about 11% (5–16%) and 10% (7–14%), respectively, which is in agreement with other studies [15–17]. As an empowerment measure for state governments, declaration of emergency could reduce *R*_*t*_ by about 13% (8–17%). Although this is not a specific NPI, we infer that a declaration of emergency not only warns people to maintain social distancing, but also provides more access to medical resources needed to prepare for the epidemic. In summary, we incorporated all nine major NPIs in a single study and further extended our understanding of the effectiveness of various NPIs. This provides comparative insights for decision making and intervention prioritizations.

All 50 US states have initiated reopening in some way since April 20th. However, some states were faced with increasing daily new cases from May to June. The findings of this study are valuable for states in making individualized plans for adjusting interventions to control the epidemic and avoid a potential second wave of cases. For states that currently have *R*_*t*_ > 1.0, relaxation is not recommended [8] and more NPIs should be re-implemented based on both current *R*_*t*_ and the differential impact of each NPI to reduce *R*_*t*_. Otherwise, infection rates could rise rapidly, resulting in more severe human health and the economic losses. For states with *R*_*t*_ < 1.0 at present or in the future, it is important to comprehensively evaluate respective NPI impacts on *R*_*t*_ (keeping *R*_*t*_ below 1.0 based on the RRs of our model), infection size, and their burden on the healthcare system. A gradual lifting plan should start with interventions that have a lower impact on *R*_*t*_ (high RRs) and then be extended to more aggressive interventions. As states began gradually relaxing interventions after April 2020, an important follow-up study concerning the impacts of the re-openings of different NPIs after April 20, 2020 is currently being conducted.

This study has several limitations. First, the three parameters for estimating *R*_*t*_, i.e., incubation time, reporting delay, and generation time, were not estimated using US data due to limited data availability. However, because incubation period estimates are similar across studies [18, 19], we believe this part of the COVID-19 virologic cycle is reasonably independent of the outbreak location. The generation time was estimated based on the data from different countries, including China, Japan, South Korea, Vietnam, Germany, US and Malaysia. This estimate was consistent with those of other studies which applied similar data to the US (mean = 5.12, SD = 4.28) [20]. The distribution of reporting delays among different districts and different time was indeed different from each other. A study in China [8] found that the empirical mean time from symptom onset to reporting was 4.9 days (SD 3.3) for Beijing, 7.6 days (4.2) for Shenzhen, and 6.3 days (4.4) for Wenzhou. Considering that the distribution from February to April in the US was not obtainable, we assume similar distribution of reporting delay between Beijing and each US state (4.9 days (SD 3.3)), which may cause some bias for the estimation of *R*_*t*_. We did a sensitivity analysis using the distribution of reporting delays in Shenzhen and Wenzhou to evaluate the possible impacts of the parameter. It showed that the core findings of this study were insensitive to this parameter. The order of the impact of different NPIs remained the same, and the quantitative values were also similar (Supplemental table 5, 6). Second, there are other confounders that have not been taken into account in evaluating the association between *R*_*t*_ and NPIs, such as climate factors and medical resources. Third, variations in the enforcement of NPIs in different states has not been taken into account, as more detailed data are required to quantify their impact. Finally, in this study, it was postulated that the impact of NPIs on *R*_*t*_ would remain fixed over time and the average *R*_*t*_ over a period was used as the response value. Incorporating the time factor into the modeling requires more data on the diversity of policies.

## Conclusions

We estimated associations between nine main NPIs and the effective reproduction number (*R*_*t*_) among 50 states in the US. We found that implementation of NPIs has substantially reduced *R*_*t*_, shedding light on their effectiveness for epidemic control. The respective impacts of different NPIs on *R*_*t*_ varied; in particular, stay-at-home decreased *R*_*t*_ to the largest extent (51, 95% CI 46–57%) and wearing (face) masks could reduce *R*_*t*_ by about 29% (15–42%). The quantitative effects also provide valuable insight for decision makers in tuning NPIs to mitigate the outbreak of other similar respiratory infectious diseases.

## Supplementary Information

**Additional file 1: Figure S1.** Symptom onset time, reporting time, incubation period, reporting delay, and generation time. a. Illustration of the relationships among the infection time, symptom onset time, reporting time, incubation period, reporting delay, and generation time (serial interval). b. Distribution of the generation time based on gamma distribution. **Figure S2.** Time varying *R*_*t*_ and inferred infection epidemic curve for the other states. The blue bars represent the daily number of infections, the orange lines show the trends of *R*_*t*_ (standard deviation less than 0.5), and the grey shading refers to the 95% confidence intervals of *R*_*t*_. The dates range from February 22 (one week before the first state emergency on February 29) to April 20. **Figure S3.** The value of *R*_*t*_ and the inferred infection number for all the 50 states on April 20.

**Additional file 2: Table S1.** Execution dates of the selected main NPIs among 50 states in the US from February 29 to April 20, and the dates of staring to relax interventions. **Table S2.** Source of information for the dates of interventions. **Table S3.** Dates of symptom onset of infector-infectee pairs. **Table S4.** Risk Ratios (RR) of each variable to the response variable ***R***_***t***_. **Table S5.** Risk Ratios (RR) of each variable to the response variable ***R***_***t***_ (Shenzhen). **Table S6.** Risk Ratios (RR) of each variable to the response variable ***R***_***t***_ (Wenzhou).

## Data Availability

The data used in the study are included in the supplementary material or in the manuscript. The code used in the study are available from the corresponding author on reasonable request. The link of the datasets used in this study are listed below. Centers for Disease Control and Prevention-Coronavirus Disease 2019 (COVID-19) (open): https://www.cdc.gov/coronavirus/2019-ncov/covid-data/covidview/index.html. Daily coronavirus cases and deaths in the US (open): https://www.worldometers.info/coronavirus/country/us/. The number of daily reported cases in the US (open): https://covidtracking.com/api. Population density by state (open): https://worldpopulationreview.com/states/. GDP by state (open): https://www.bea.gov/data/gdp/gdp-state. Median age by state (open): http://www.statsamerica.org/sip/rank_list.aspx?rank_label=pop46&ct=S09. STATSAMERICA-Data Source: US Census Bureau.

## Abbreviations

COVID-19: Coronavirus disease 2019
US: United States
NPIs: Non-pharmaceutical public health interventions
*R*_*t*_: The effective reproduction number
RR: Risk ratios
GLM: Generalized linear model
SARS-COV-2: Severe acute respiratory syndrome coronavirus 2
NY: New York
