# Supplementary material for: Differential impact of non-pharmaceutical public health interventions on COVID-19 epidemics in the United States
Source: BMC Public Health. 2021 May 21;21:965. doi: 10.1186/s12889-021-10950-2 (PMC8139542; doi:10.1186/s12889-021-10950-2)
Supplement: Supplementary file 1 — Additional file 1: Figure S1. Symptom onset time, reporting time, incubation period, reporting delay, and generation time. a. Illustration of the relationships among the infection time, symptom onset time, reporting time, incubation period, reporting delay, and generation time (serial interval). b. Distribution of the generation time based on gamma distribution. Figure S2. Time varying Rt and inferred infection epidemic curve for the other states. The blue bars represent the daily number of infections, the orange lines show the trends of Rt (standard deviation less than 0.5), and the grey shading refers to the 95% confidence intervals of Rt. The dates range from February 22 (one week before the first state emergency on February 29) to April 20. Figure S3. The value of Rt and the inferred infection number for all the 50 states on April 20. [file 12889_2021_10950_MOESM1_ESM.docx]

**Differential impact of non-pharmaceutical public health interventions on COVID-19 epidemics in the United States**

- BMC public health

Xiaoshuang Liu^1,*^; Xiao Xu^1,*^; Guanqiao Li^2,*^; Xian Xu^1^; Yuyao Sun^1^; Fei Wang^3^; Xuanling Shi^2^; Xiang Li^1,+^; Guotong Xie^1,4,5+^; Linqi Zhang^2,+^

^*^ Contributed equally

^+^ To whom correspondence may be addressed.

^1^Ping An Healthcare Technology, Beijing, China;

^2^Center for Global Health and Infectious Diseases, School of Medicine, Tsinghua University, Beijing, China;

^3^Department of Healthcare Policy and Research. Weill Cornell Medicine. Cornell University, New York, USA

^4^Ping An Health Cloud Company Limited, Beijing, China;

^5^Ping An International Smart City Technology Co., Ltd., Beijing, China;

Corresponding author: Linqi Zhang, [zhanglinqi@tsinghua.edu.cn](mailto:zhanglinqi@tsinghua.edu.cn), 86-10-62788131.

**Supplemental Figures**

**Fig. S1 Symptom onset time, reporting time, incubation period, reporting delay, and generation time.** a. Illustration of the relationships among the infection time, symptom onset time, reporting time, incubation period, reporting delay, and generation time (serial interval). b. Distribution of the generation time based on gamma distribution.


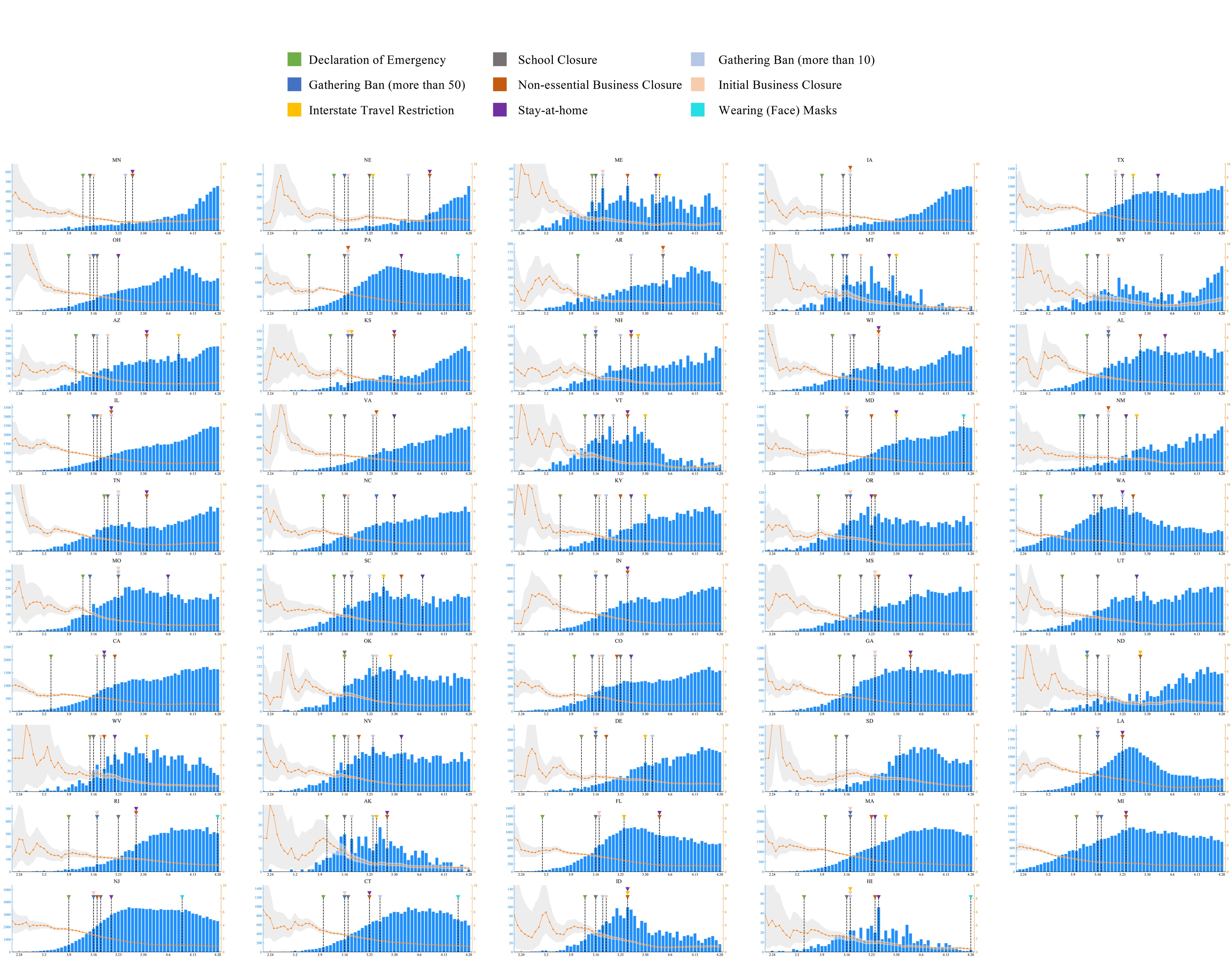


**Fig. S2 Time varying** $\boldsymbol{R}_{\boldsymbol{t}}$ **and inferred infection epidemic curve for the other states**. The blue bars represent the daily number of infections, the orange lines show the trends of $R_{t}$ (standard deviation less than 0.5), and the grey shading refers to the 95% confidence intervals of $R_{t}$. The dates range from February 22 (one week before the first state emergency on February 29) to April 20.

**Fig. S3 The value of** $\boldsymbol{R}_{\boldsymbol{t}}$ **and the inferred infection number for all the 50 states on April 20.**
