# Supplementary material for: Differential impact of non-pharmaceutical public health interventions on COVID-19 epidemics in the United States
Source: BMC Public Health. 2021 May 21;21:965. doi: 10.1186/s12889-021-10950-2 (PMC8139542; doi:10.1186/s12889-021-10950-2)
Supplement: Supplementary file 2 — Additional file 2: Table S1. Execution dates of the selected main NPIs among 50 states in the US from February 29 to April 20, and the dates of staring to relax interventions. Table S2. Source of information for the dates of interventions. Table S3. Dates of symptom onset of infector-infectee pairs. Table S4. Risk Ratios (RR) of each variable to the response variable Rt. Table S5. Risk Ratios (RR) of each variable to the response variable Rt (Shenzhen). Table S6. Risk Ratios (RR) of each variable to the response variable Rt (Wenzhou). [file 12889_2021_10950_MOESM2_ESM.docx]

**Supplementary Table 1. Execution dates of the selected main NPIs among 50 states in the US from February 29 to April 20, and the dates of staring to relax interventions.**

| **No.** | **State** | **Abbr.** | **Declaration of Emergency** | **School Closure** | **Gathering Ban**  **(more than 10)** | **Gathering Ban**  **(more than 50)** | **Initial Business Closure** | **Non-essential Business Closure** | **Interstate Travel Restriction** | **Stay-at-home** | **Wearing (Face) Masks** |
| --- | --- | --- | --- | --- | --- | --- | --- | --- | --- | --- | --- |
| 1 | Alabama | AL | 2020/3/13 | 2020/3/19 | NA^a^ | 2020/3/19 | 2020/3/19 | 2020/3/28 | NA | 2020/4/4 | NA |
| 2 | Alaska | AK | 2020/3/11 | 2020/3/16 | 2020/3/24 | NA | 2020/3/18 | 2020/3/28 | 2020/3/25 | 2020/3/28 | NA |
| 3 | Arizona | AZ | 2020/3/11 | 2020/3/16 | 2020/3/17 | NA | 2020/3/20 | 2020/3/31 | 2020/4/9 | 2020/3/31 | NA |
| 4 | Arkansas | AR | 2020/3/11 | 2020/4/4 | 2020/3/26 | NA | 2020/4/4 | 2020/4/4 | NA | NA | NA |
| 5 | California | CA | 2020/3/4 | 2020/3/19 | NA | NA | 2020/3/17 | 2020/3/19 | NA | 2020/3/19 | NA |
| 6 | Colorado | CO | 2020/3/10 | 2020/3/23 | 2020/3/18 | 2020/3/15 | 2020/3/17 | 2020/3/22 | NA | 2020/3/26 | NA |
| 7 | Connecticut | CT | 2020/3/10 | 2020/3/17 | 2020/3/23 | 2020/3/16 | 2020/3/16 | 2020/3/23 | NA | 2020/3/23 | 2020/4/17 |
| 8 | Delaware | DE | 2020/3/12 | 2020/3/16 | 2020/4/1 | 2020/3/16 | 2020/3/16 | 2020/3/19 | 2020/3/30 | NA | NA |
| 9 | Florida | FL | 2020/3/1 | 2020/3/16 | 2020/3/17 | NA | 2020/3/17 | 2020/4/3 | 2020/3/24 | 2020/4/3 | NA |
| 10 | Georgia | GA | 2020/3/14 | 2020/3/18 | 2020/3/24 | NA | 2020/3/24 | 2020/4/3 | NA | 2020/4/3 | NA |
| 11 | Hawaii | HI | 2020/3/4 | 2020/3/16 | 2020/3/17 | NA | 2020/3/17 | 2020/3/24 | 2020/3/17 | 2020/3/25 | 2020/4/20 |
| 12 | Idaho | ID | 2020/3/13 | 2020/3/16 | 2020/3/18 | NA | 2020/3/19 | 2020/3/25 | 2020/3/25 | 2020/3/25 | NA |
| 13 | Illinois | IL | 2020/3/9 | 2020/3/17 | 2020/3/21 | 2020/3/16 | 2020/3/18 | 2020/3/21 | NA | 2020/3/21 | NA |
| 14 | Indiana | IN | 2020/3/6 | 2020/3/19 | 2020/3/25 | 2020/3/16 | 2020/3/16 | 2020/3/25 | NA | 2020/3/25 | NA |
| 15 | Iowa | IA | 2020/3/9 | 2020/3/15 | 2020/3/17 | NA | 2020/3/17 | 2020/3/17 | NA | NA | NA |
| 16 | Kansas | KS | 2020/3/12 | 2020/3/18 | NA | 2020/3/17 | 2020/3/17 | 2020/3/30 | 2020/3/18 | 2020/3/30 | NA |
| 17 | Kentucky | KY | 2020/3/6 | 2020/3/16 | 2020/3/19 | NA | 2020/3/17 | 2020/3/23 | 2020/3/30 | 2020/3/26 | NA |
| 18 | Louisiana | LA | 2020/3/11 | 2020/3/16 | NA | 2020/3/16 | 2020/3/16 | 2020/3/23 | NA | 2020/3/23 | NA |
| 19 | Maine | ME | 2020/3/15 | 2020/3/16 | 2020/3/18 | NA | 2020/3/18 | 2020/3/25 | 2020/4/3 | 2020/4/2 | NA |
| 20 | Maryland | MD | 2020/3/5 | 2020/3/16 | NA | 2020/3/16 | 2020/3/16 | 2020/3/23 | 2020/3/30 | 2020/3/30 | 2020/4/18 |
| 21 | Massachusetts | MA | 2020/3/10 | 2020/3/17 | NA | 2020/3/17 | 2020/3/17 | 2020/3/23 | 2020/3/27 | 2020/3/24 | NA |
| 22 | Michigan | MI | 2020/3/10 | 2020/3/16 | NA | 2020/3/17 | 2020/3/16 | 2020/3/24 | NA | 2020/3/24 | NA |
| 23 | Minnesota | MN | 2020/3/13 | 2020/3/15 | 2020/3/25 | NA | 2020/3/16 | 2020/3/27 | NA | 2020/3/27 | NA |
| 24 | Mississippi | MS | 2020/3/14 | 2020/3/20 | 2020/3/24 | NA | 2020/3/24 | 2020/3/25 | NA | 2020/4/3 | NA |
| 25 | Missouri | MO | 2020/3/13 | 2020/3/23 | 2020/3/23 | 2020/3/15 | 2020/3/23 | NA | NA | 2020/4/6 | NA |
| 26 | Montana | MT | 2020/3/12 | 2020/3/16 | NA | 2020/3/15 | 2020/3/20 | NA | 2020/3/30 | 2020/3/28 | NA |
| 27 | Nebraska | NE | 2020/3/13 | 2020/3/23 | 2020/4/3 | 2020/3/16 | 2020/3/17 | 2020/4/9 | 2020/3/24 | 2020/4/9 | NA |
| 28 | Nevada | NV | 2020/3/13 | 2020/3/16 | 2020/3/24 | NA | 2020/3/17 | 2020/3/20 | NA | 2020/4/1 | NA |
| 29 | New Hampshire | NH | 2020/3/13 | 2020/3/16 | 2020/3/23 | 2020/3/16 | 2020/3/16 | 2020/3/26 | 2020/3/28 | 2020/3/26 | NA |
| 30 | New Jersey | NJ | 2020/3/9 | 2020/3/18 | NA | 2020/3/16 | 2020/3/16 | 2020/3/17 | NA | 2020/3/21 | 2020/4/10 |
| 31 | New Mexico | NM | 2020/3/11 | 2020/3/16 | 2020/3/19 | 2020/3/12 | 2020/3/19 | 2020/3/19 | 2020/3/27 | 2020/3/24 | NA |
| 32 | New York | NY | 2020/3/7 | 2020/3/18 | NA | 2020/3/16 | 2020/3/16 | 2020/3/20 | NA | 2020/3/22 | 2020/4/15 |
| 33 | North Carolina | NC | 2020/3/10 | 2020/3/16 | NA | 2020/3/25 | 2020/3/17 | NA | NA | 2020/3/30 | NA |
| 34 | North Dakota | ND | 2020/3/13 | 2020/3/16 | NA | 2020/3/13 | 2020/3/19 | 2020/3/28 | 2020/3/28 | NA | NA |
| 35 | Ohio | OH | 2020/3/9 | 2020/3/17 | NA | 2020/3/16 | 2020/3/15 | NA | NA | 2020/3/23 | NA |
| 36 | Oklahoma | OK | 2020/3/16 | 2020/3/16 | 2020/3/24 | NA | 2020/3/25 | NA | 2020/3/29 | NA | NA |
| 37 | Oregon | OR | 2020/3/8 | 2020/3/16 | NA | 2020/3/17 | 2020/3/17 | 2020/3/23 | NA | 2020/3/23 | NA |
| 38 | Pennsylvania | PA | 2020/3/6 | 2020/3/16 | 2020/3/17 | NA | 2020/3/17 | 2020/3/17 | NA | 2020/4/1 | 2020/4/17 |
| 39 | Rhode Island | RI | 2020/3/9 | 2020/3/23 | 2020/3/28 | 2020/3/17 | 2020/3/17 | 2020/3/28 | NA | 2020/3/28 | 2020/4/20 |
| 40 | South Carolina | SC | 2020/3/13 | 2020/3/16 | 2020/3/23 | 2020/3/18 | 2020/3/18 | 2020/4/1 | 2020/3/27 | 2020/4/7 | NA |
| 41 | South Dakota | SD | 2020/3/13 | 2020/3/16 | 2020/3/31 | NA | NA | NA | NA | NA | NA |
| 42 | Tennessee | TN | 2020/3/19 | 2020/3/20 | 2020/3/23 | NA | 2020/3/23 | 2020/3/31 | NA | 2020/3/31 | NA |
| 43 | Texas | TX | 2020/3/13 | 2020/3/23 | 2020/3/21 | NA | 2020/3/21 | NA | 2020/3/26 | 2020/4/2 | NA |
| 44 | Utah | UT | 2020/3/6 | 2020/3/16 | NA | NA | NA | NA | NA | 2020/3/27 | NA |
| 45 | Vermont | VT | 2020/3/13 | 2020/3/18 | 2020/3/21 | 2020/3/16 | 2020/3/17 | 2020/3/25 | 2020/3/30 | 2020/3/25 | NA |
| 46 | Virginia | VA | 2020/3/12 | 2020/3/16 | 2020/3/24 | NA | 2020/3/25 | 2020/3/25 | NA | 2020/3/30 | NA |
| 47 | Washington | WA | 2020/2/29 | 2020/3/17 | 2020/3/23 | 2020/3/15 | 2020/3/16 | 2020/3/23 | NA | 2020/3/23 | NA |
| 48 | West Virginia | WV | 2020/3/15 | 2020/3/16 | NA | NA | 2020/3/18 | 2020/3/19 | 2020/3/31 | 2020/3/22 | NA |
| 49 | Wisconsin | WI | 2020/3/12 | 2020/3/18 | 2020/3/17 | NA | 2020/3/25 | 2020/3/25 | NA | 2020/3/25 | NA |
| 50 | Wyoming | WY | 2020/3/13 | 2020/3/16 | 2020/4/3 | NA | 2020/3/19 | NA | NA | NA | NA |

a, NA represents that the state has not executed the intervention.

**Supplementary Table 2. Source of information for the dates of interventions.**

| **No.** | **Source of information** |
| --- | --- |
| 1 | [https://governor.alabama.gov/newsroom/2020/03/state-of-emergency-coronavirus-covid-19/ https://governor.alabama.gov/newsroom/2020/03/governor-ivey-issues-statement-on-statewide-public-health-order/](https://governor.alabama.gov/newsroom/2020/03/state-of-emergency-coronavirus-covid-19/https://governor.alabama.gov/newsroom/2020/03/governor-ivey-issues-statement-on-statewide-public-health-order/https://governor.alabama.gov/newsroom/2020/03/supplemental-state-of-emergency-coronavirus-covid-19/https://governor.alabama.gov/assets/2020/03/Amended-Statewide-Social-Distancing-SHO-Order-3.27.2020-FINAL.pdf)  [https://governor.alabama.gov/newsroom/2020/03/supplemental-state-of-emergency-coronavirus-covid-19/](https://governor.alabama.gov/newsroom/2020/03/state-of-emergency-coronavirus-covid-19/https://governor.alabama.gov/newsroom/2020/03/governor-ivey-issues-statement-on-statewide-public-health-order/https://governor.alabama.gov/newsroom/2020/03/supplemental-state-of-emergency-coronavirus-covid-19/https://governor.alabama.gov/assets/2020/03/Amended-Statewide-Social-Distancing-SHO-Order-3.27.2020-FINAL.pdf)  [https://governor.alabama.gov/assets/2020/03/Amended-Statewide-Social-Distancing-SHO-Order-3.27.2020-FINAL.pdf](https://governor.alabama.gov/newsroom/2020/03/state-of-emergency-coronavirus-covid-19/https://governor.alabama.gov/newsroom/2020/03/governor-ivey-issues-statement-on-statewide-public-health-order/https://governor.alabama.gov/newsroom/2020/03/supplemental-state-of-emergency-coronavirus-covid-19/https://governor.alabama.gov/assets/2020/03/Amended-Statewide-Social-Distancing-SHO-Order-3.27.2020-FINAL.pdf)  <https://www.edweek.org/ew/section/multimedia/map-coronavirus-and-school-closures.html>  <https://www.nytimes.com/interactive/2020/us/coronavirus-stay-at-home-order.html> |
| 2 | [https://gov.alaska.gov/wp-content/uploads/sites/2/03172020-SOA-COVID-19-Health-Mandate-003.pdf](https://gov.alaska.gov/wp-content/uploads/sites/2/03172020-SOA-COVID-19-Health-Mandate-003.pdfhttps://covid19.alaska.gov/health-mandates/https://gov.alaska.gov/wp-content/uploads/sites/2/03272020-SOA-COVID-19-Health-Mandate-011.pdf)  [https://covid19.alaska.gov/health-mandates/](https://gov.alaska.gov/wp-content/uploads/sites/2/03172020-SOA-COVID-19-Health-Mandate-003.pdfhttps://covid19.alaska.gov/health-mandates/https://gov.alaska.gov/wp-content/uploads/sites/2/03272020-SOA-COVID-19-Health-Mandate-011.pdf)  [https://gov.alaska.gov/wp-content/uploads/sites/2/03272020-SOA-COVID-19-Health-Mandate-011.pdf](https://gov.alaska.gov/wp-content/uploads/sites/2/03172020-SOA-COVID-19-Health-Mandate-003.pdfhttps://covid19.alaska.gov/health-mandates/https://gov.alaska.gov/wp-content/uploads/sites/2/03272020-SOA-COVID-19-Health-Mandate-011.pdf)  <https://ballotpedia.org/Travel_restrictions_issued_by_states_in_response_to_the_coronavirus_(COVID-19)_pandemic,_2020>  <https://www.edweek.org/ew/section/multimedia/map-coronavirus-and-school-closures.html>  <https://www.nytimes.com/interactive/2020/us/coronavirus-stay-at-home-order.html> |
| 3 | [https://azgovernor.gov/governor/news/2020/03/governor-doug-ducey-issues-declaration-emergency-executive-order-combat](https://azgovernor.gov/governor/news/2020/03/governor-doug-ducey-issues-declaration-emergency-executive-order-combathttps://azgovernor.gov/governor/news/2020/03/governor-ducey-announces-latest-covid-19-actionshttps://www.cnbc.com/2020/03/15/california-governor-calls-for-closure-of-all-bars-and-wineries-home-isolation-of-seniors.html)  [https://azgovernor.gov/governor/news/2020/03/governor-ducey-announces-latest-covid-19-actions](https://azgovernor.gov/governor/news/2020/03/governor-doug-ducey-issues-declaration-emergency-executive-order-combathttps://azgovernor.gov/governor/news/2020/03/governor-ducey-announces-latest-covid-19-actionshttps://www.cnbc.com/2020/03/15/california-governor-calls-for-closure-of-all-bars-and-wineries-home-isolation-of-seniors.html)  [https://www.cnbc.com/2020/03/15/california-governor-calls-for-closure-of-all-bars-and-wineries-home-isolation-of-seniors.html](https://azgovernor.gov/governor/news/2020/03/governor-doug-ducey-issues-declaration-emergency-executive-order-combathttps://azgovernor.gov/governor/news/2020/03/governor-ducey-announces-latest-covid-19-actionshttps://www.cnbc.com/2020/03/15/california-governor-calls-for-closure-of-all-bars-and-wineries-home-isolation-of-seniors.html)  <https://ballotpedia.org/Travel_restrictions_issued_by_states_in_response_to_the_coronavirus_(COVID-19)_pandemic,_2020>  <https://www.edweek.org/ew/section/multimedia/map-coronavirus-and-school-closures.html>  <https://www.nytimes.com/interactive/2020/us/coronavirus-stay-at-home-order.html> |
| 4 | [https://governor.arkansas.gov/images/uploads/executiveOrders/EO_20-03.__1.pdf](https://governor.arkansas.gov/images/uploads/executiveOrders/EO_20-03.__1.pdfhttps://governor.arkansas.gov/images/uploads/executiveOrders/EO_20-13._.pdfhttps://governor.arkansas.gov/images/uploads/executiveOrders/EO_20-10._.pdfhttps://governor.arkansas.gov/images/uploads/executiveOrders/EO_20-13._.pdfhttps://governor.arkansas.gov/images/uploads/executiveOrders/EO_20-13._.pdf)  [https://governor.arkansas.gov/images/uploads/executiveOrders/EO_20-13._.pdf](https://governor.arkansas.gov/images/uploads/executiveOrders/EO_20-03.__1.pdfhttps://governor.arkansas.gov/images/uploads/executiveOrders/EO_20-13._.pdfhttps://governor.arkansas.gov/images/uploads/executiveOrders/EO_20-10._.pdfhttps://governor.arkansas.gov/images/uploads/executiveOrders/EO_20-13._.pdfhttps://governor.arkansas.gov/images/uploads/executiveOrders/EO_20-13._.pdf)  [https://governor.arkansas.gov/images/uploads/executiveOrders/EO_20-10._.pdf](https://governor.arkansas.gov/images/uploads/executiveOrders/EO_20-03.__1.pdfhttps://governor.arkansas.gov/images/uploads/executiveOrders/EO_20-13._.pdfhttps://governor.arkansas.gov/images/uploads/executiveOrders/EO_20-10._.pdfhttps://governor.arkansas.gov/images/uploads/executiveOrders/EO_20-13._.pdfhttps://governor.arkansas.gov/images/uploads/executiveOrders/EO_20-13._.pdf)  [https://governor.arkansas.gov/images/uploads/executiveOrders/EO_20-13._.pdf](https://governor.arkansas.gov/images/uploads/executiveOrders/EO_20-03.__1.pdfhttps://governor.arkansas.gov/images/uploads/executiveOrders/EO_20-13._.pdfhttps://governor.arkansas.gov/images/uploads/executiveOrders/EO_20-10._.pdfhttps://governor.arkansas.gov/images/uploads/executiveOrders/EO_20-13._.pdfhttps://governor.arkansas.gov/images/uploads/executiveOrders/EO_20-13._.pdf)  [https://governor.arkansas.gov/images/uploads/executiveOrders/EO_20-13._.pdf](https://governor.arkansas.gov/images/uploads/executiveOrders/EO_20-03.__1.pdfhttps://governor.arkansas.gov/images/uploads/executiveOrders/EO_20-13._.pdfhttps://governor.arkansas.gov/images/uploads/executiveOrders/EO_20-10._.pdfhttps://governor.arkansas.gov/images/uploads/executiveOrders/EO_20-13._.pdfhttps://governor.arkansas.gov/images/uploads/executiveOrders/EO_20-13._.pdf)  <https://www.edweek.org/ew/section/multimedia/map-coronavirus-and-school-closures.html> |
| 5 | [https://www.gov.ca.gov/2020/03/04/governor-newsom-declares-state-of-emergency-to-help-state-prepare-for-broader-spread-of-covid-19/](https://www.gov.ca.gov/2020/03/04/governor-newsom-declares-state-of-emergency-to-help-state-prepare-for-broader-spread-of-covid-19/https://www.gov.ca.gov/2020/03/11/california-public-health-experts-mass-gatherings-should-be-postponed-or-canceled-statewide-to-slow-the-spread-of-covid-19/https://www.gov.ca.gov/california-takes-action-to-combat-covid-19/)  [https://www.gov.ca.gov/2020/03/11/california-public-health-experts-mass-gatherings-should-be-postponed-or-canceled-statewide-to-slow-the-spread-of-covid-19/](https://www.gov.ca.gov/2020/03/04/governor-newsom-declares-state-of-emergency-to-help-state-prepare-for-broader-spread-of-covid-19/https://www.gov.ca.gov/2020/03/11/california-public-health-experts-mass-gatherings-should-be-postponed-or-canceled-statewide-to-slow-the-spread-of-covid-19/https://www.gov.ca.gov/california-takes-action-to-combat-covid-19/)  [https://www.gov.ca.gov/california-takes-action-to-combat-covid-19/](https://www.gov.ca.gov/2020/03/04/governor-newsom-declares-state-of-emergency-to-help-state-prepare-for-broader-spread-of-covid-19/https://www.gov.ca.gov/2020/03/11/california-public-health-experts-mass-gatherings-should-be-postponed-or-canceled-statewide-to-slow-the-spread-of-covid-19/https://www.gov.ca.gov/california-takes-action-to-combat-covid-19/)  <https://www.edweek.org/ew/section/multimedia/map-coronavirus-and-school-closures.html> |
| 6 | [https://www.colorado.gov/governor/news/cdphe-recommends-colorado-follow-cdc-guidance-cancel-or-postpone-person-events-50-people-or](https://www.colorado.gov/governor/news/cdphe-recommends-colorado-follow-cdc-guidance-cancel-or-postpone-person-events-50-people-orhttps://www.colorado.gov/governor/news/state-health-department-issues-public-health-order-closing-colorado-bars-restaurants-gymshttps://www.colorado.gov/pacific/sites/default/files/atoms/files/Bars%20Restaurants%20PH%20order.pdf)  [https://www.colorado.gov/governor/news/state-health-department-issues-public-health-order-closing-colorado-bars-restaurants-gyms](https://www.colorado.gov/governor/news/cdphe-recommends-colorado-follow-cdc-guidance-cancel-or-postpone-person-events-50-people-orhttps://www.colorado.gov/governor/news/state-health-department-issues-public-health-order-closing-colorado-bars-restaurants-gymshttps://www.colorado.gov/pacific/sites/default/files/atoms/files/Bars%20Restaurants%20PH%20order.pdf)  [https://www.colorado.gov/pacific/sites/default/files/atoms/files/Bars%20Restaurants%20PH%20order.pdf](https://www.colorado.gov/governor/news/cdphe-recommends-colorado-follow-cdc-guidance-cancel-or-postpone-person-events-50-people-orhttps://www.colorado.gov/governor/news/state-health-department-issues-public-health-order-closing-colorado-bars-restaurants-gymshttps://www.colorado.gov/pacific/sites/default/files/atoms/files/Bars%20Restaurants%20PH%20order.pdf)  <https://www.edweek.org/ew/section/multimedia/map-coronavirus-and-school-closures.html>  <https://www.nytimes.com/interactive/2020/us/coronavirus-stay-at-home-order.html> |
| 7 | [https://portal.ct.gov/Office-of-the-Governor/News/Press-Releases/2020/03-2020/Governor-Lamont-Coronavirus-Update-March-10-2020-3PM](https://portal.ct.gov/Office-of-the-Governor/News/Press-Releases/2020/03-2020/Governor-Lamont-Coronavirus-Update-March-10-2020-3PMhttps://portal.ct.gov/-/media/Office-of-the-Governor/Executive-Orders/Lamont-Executive-Orders/Executive-Order-No-7H.pdf)  [https://portal.ct.gov/-/media/Office-of-the-Governor/Executive-Orders/Lamont-Executive-Orders/Executive-Order-No-7H.pdf](https://portal.ct.gov/Office-of-the-Governor/News/Press-Releases/2020/03-2020/Governor-Lamont-Coronavirus-Update-March-10-2020-3PMhttps://portal.ct.gov/-/media/Office-of-the-Governor/Executive-Orders/Lamont-Executive-Orders/Executive-Order-No-7H.pdf)  <https://www.edweek.org/ew/section/multimedia/map-coronavirus-and-school-closures.html>  https://www.nbcconnecticut.com/news/coronavirus/masks-or-cloth-face-coverings-required-in-public-in-conn-starting-tonight-to-slow-covid-19/2258053/ |
| 8 | [https://news.delaware.gov/2020/03/12/governor-carney-declares-state-of-emergency-to-prepare-for-spread-of-coronavirus-covid-19/](https://news.delaware.gov/2020/03/12/governor-carney-declares-state-of-emergency-to-prepare-for-spread-of-coronavirus-covid-19/https://news.delaware.gov/2020/03/13/governor-carney-directs-two-week-closure-of-delaware-public-schools/https://news.delaware.gov/2020/03/12/governor-carney-declares-state-of-emergency-to-prepare-for-spread-of-coronavirus-covid-19/https://news.delaware.gov/2020/03/16/governor-carney-limits-restaurants-to-take-out-and-delivery/https://news.delaware.gov/2020/04/01/governor-carney-restricts-gatherings-requires-businesses-to-strictly-comply-with-social-distancing/https://news.delaware.gov/2020/03/16/governor-carney-limits-restaurants-to-take-out-and-delivery/https://news.delaware.gov/2020/03/18/governor-carney-issues-second-update-to-emergency-declaration/https://news.delaware.gov/2020/03/22/governor-carney-issues-stay-at-home-order-for-delawareans/)  [https://news.delaware.gov/2020/03/13/governor-carney-directs-two-week-closure-of-delaware-public-schools/](https://news.delaware.gov/2020/03/12/governor-carney-declares-state-of-emergency-to-prepare-for-spread-of-coronavirus-covid-19/https://news.delaware.gov/2020/03/13/governor-carney-directs-two-week-closure-of-delaware-public-schools/https://news.delaware.gov/2020/03/12/governor-carney-declares-state-of-emergency-to-prepare-for-spread-of-coronavirus-covid-19/https://news.delaware.gov/2020/03/16/governor-carney-limits-restaurants-to-take-out-and-delivery/https://news.delaware.gov/2020/04/01/governor-carney-restricts-gatherings-requires-businesses-to-strictly-comply-with-social-distancing/https://news.delaware.gov/2020/03/16/governor-carney-limits-restaurants-to-take-out-and-delivery/https://news.delaware.gov/2020/03/18/governor-carney-issues-second-update-to-emergency-declaration/https://news.delaware.gov/2020/03/22/governor-carney-issues-stay-at-home-order-for-delawareans/)  [https://news.delaware.gov/2020/03/12/governor-carney-declares-state-of-emergency-to-prepare-for-spread-of-coronavirus-covid-19/](https://news.delaware.gov/2020/03/12/governor-carney-declares-state-of-emergency-to-prepare-for-spread-of-coronavirus-covid-19/https://news.delaware.gov/2020/03/13/governor-carney-directs-two-week-closure-of-delaware-public-schools/https://news.delaware.gov/2020/03/12/governor-carney-declares-state-of-emergency-to-prepare-for-spread-of-coronavirus-covid-19/https://news.delaware.gov/2020/03/16/governor-carney-limits-restaurants-to-take-out-and-delivery/https://news.delaware.gov/2020/04/01/governor-carney-restricts-gatherings-requires-businesses-to-strictly-comply-with-social-distancing/https://news.delaware.gov/2020/03/16/governor-carney-limits-restaurants-to-take-out-and-delivery/https://news.delaware.gov/2020/03/18/governor-carney-issues-second-update-to-emergency-declaration/https://news.delaware.gov/2020/03/22/governor-carney-issues-stay-at-home-order-for-delawareans/)  [https://news.delaware.gov/2020/03/16/governor-carney-limits-restaurants-to-take-out-and-delivery/](https://news.delaware.gov/2020/03/12/governor-carney-declares-state-of-emergency-to-prepare-for-spread-of-coronavirus-covid-19/https://news.delaware.gov/2020/03/13/governor-carney-directs-two-week-closure-of-delaware-public-schools/https://news.delaware.gov/2020/03/12/governor-carney-declares-state-of-emergency-to-prepare-for-spread-of-coronavirus-covid-19/https://news.delaware.gov/2020/03/16/governor-carney-limits-restaurants-to-take-out-and-delivery/https://news.delaware.gov/2020/04/01/governor-carney-restricts-gatherings-requires-businesses-to-strictly-comply-with-social-distancing/https://news.delaware.gov/2020/03/16/governor-carney-limits-restaurants-to-take-out-and-delivery/https://news.delaware.gov/2020/03/18/governor-carney-issues-second-update-to-emergency-declaration/https://news.delaware.gov/2020/03/22/governor-carney-issues-stay-at-home-order-for-delawareans/)  [https://news.delaware.gov/2020/04/01/governor-carney-restricts-gatherings-requires-businesses-to-strictly-comply-with-social-distancing/](https://news.delaware.gov/2020/03/12/governor-carney-declares-state-of-emergency-to-prepare-for-spread-of-coronavirus-covid-19/https://news.delaware.gov/2020/03/13/governor-carney-directs-two-week-closure-of-delaware-public-schools/https://news.delaware.gov/2020/03/12/governor-carney-declares-state-of-emergency-to-prepare-for-spread-of-coronavirus-covid-19/https://news.delaware.gov/2020/03/16/governor-carney-limits-restaurants-to-take-out-and-delivery/https://news.delaware.gov/2020/04/01/governor-carney-restricts-gatherings-requires-businesses-to-strictly-comply-with-social-distancing/https://news.delaware.gov/2020/03/16/governor-carney-limits-restaurants-to-take-out-and-delivery/https://news.delaware.gov/2020/03/18/governor-carney-issues-second-update-to-emergency-declaration/https://news.delaware.gov/2020/03/22/governor-carney-issues-stay-at-home-order-for-delawareans/)  [https://news.delaware.gov/2020/03/16/governor-carney-limits-restaurants-to-take-out-and-delivery/](https://news.delaware.gov/2020/03/12/governor-carney-declares-state-of-emergency-to-prepare-for-spread-of-coronavirus-covid-19/https://news.delaware.gov/2020/03/13/governor-carney-directs-two-week-closure-of-delaware-public-schools/https://news.delaware.gov/2020/03/12/governor-carney-declares-state-of-emergency-to-prepare-for-spread-of-coronavirus-covid-19/https://news.delaware.gov/2020/03/16/governor-carney-limits-restaurants-to-take-out-and-delivery/https://news.delaware.gov/2020/04/01/governor-carney-restricts-gatherings-requires-businesses-to-strictly-comply-with-social-distancing/https://news.delaware.gov/2020/03/16/governor-carney-limits-restaurants-to-take-out-and-delivery/https://news.delaware.gov/2020/03/18/governor-carney-issues-second-update-to-emergency-declaration/https://news.delaware.gov/2020/03/22/governor-carney-issues-stay-at-home-order-for-delawareans/)  [https://news.delaware.gov/2020/03/18/governor-carney-issues-second-update-to-emergency-declaration/](https://news.delaware.gov/2020/03/12/governor-carney-declares-state-of-emergency-to-prepare-for-spread-of-coronavirus-covid-19/https://news.delaware.gov/2020/03/13/governor-carney-directs-two-week-closure-of-delaware-public-schools/https://news.delaware.gov/2020/03/12/governor-carney-declares-state-of-emergency-to-prepare-for-spread-of-coronavirus-covid-19/https://news.delaware.gov/2020/03/16/governor-carney-limits-restaurants-to-take-out-and-delivery/https://news.delaware.gov/2020/04/01/governor-carney-restricts-gatherings-requires-businesses-to-strictly-comply-with-social-distancing/https://news.delaware.gov/2020/03/16/governor-carney-limits-restaurants-to-take-out-and-delivery/https://news.delaware.gov/2020/03/18/governor-carney-issues-second-update-to-emergency-declaration/https://news.delaware.gov/2020/03/22/governor-carney-issues-stay-at-home-order-for-delawareans/)  [https://news.delaware.gov/2020/03/22/governor-carney-issues-stay-at-home-order-for-delawareans/](https://news.delaware.gov/2020/03/12/governor-carney-declares-state-of-emergency-to-prepare-for-spread-of-coronavirus-covid-19/https://news.delaware.gov/2020/03/13/governor-carney-directs-two-week-closure-of-delaware-public-schools/https://news.delaware.gov/2020/03/12/governor-carney-declares-state-of-emergency-to-prepare-for-spread-of-coronavirus-covid-19/https://news.delaware.gov/2020/03/16/governor-carney-limits-restaurants-to-take-out-and-delivery/https://news.delaware.gov/2020/04/01/governor-carney-restricts-gatherings-requires-businesses-to-strictly-comply-with-social-distancing/https://news.delaware.gov/2020/03/16/governor-carney-limits-restaurants-to-take-out-and-delivery/https://news.delaware.gov/2020/03/18/governor-carney-issues-second-update-to-emergency-declaration/https://news.delaware.gov/2020/03/22/governor-carney-issues-stay-at-home-order-for-delawareans/)  <https://ballotpedia.org/Travel_restrictions_issued_by_states_in_response_to_the_coronavirus_(COVID-19)_pandemic,_2020>  <https://www.edweek.org/ew/section/multimedia/map-coronavirus-and-school-closures.html> |
| 9 | [https://www.flgov.com/wp-content/uploads/orders/2020/EO_20-51.pdf](https://www.flgov.com/wp-content/uploads/orders/2020/EO_20-51.pdfhttps://www.flgov.com/wp-content/uploads/orders/2020/EO_20-68.pdf)  [https://www.flgov.com/wp-content/uploads/orders/2020/EO_20-68.pdf](https://www.flgov.com/wp-content/uploads/orders/2020/EO_20-51.pdfhttps://www.flgov.com/wp-content/uploads/orders/2020/EO_20-68.pdf)  <https://ballotpedia.org/Travel_restrictions_issued_by_states_in_response_to_the_coronavirus_(COVID-19)_pandemic,_2020>  <https://www.edweek.org/ew/section/multimedia/map-coronavirus-and-school-closures.html>  <https://www.nytimes.com/interactive/2020/us/coronavirus-stay-at-home-order.html> |
| 10 | [https://gov.georgia.gov/press-releases/2020-03-16/kemp-declares-public-health-state-emergency](https://gov.georgia.gov/press-releases/2020-03-16/kemp-declares-public-health-state-emergencyhttps://gov.georgia.gov/press-releases/2020-03-23/gov-kemp-issues-new-executive-orders-provides-covid-19-update)  [https://gov.georgia.gov/press-releases/2020-03-23/gov-kemp-issues-new-executive-orders-provides-covid-19-update](https://gov.georgia.gov/press-releases/2020-03-16/kemp-declares-public-health-state-emergencyhttps://gov.georgia.gov/press-releases/2020-03-23/gov-kemp-issues-new-executive-orders-provides-covid-19-update)  <https://www.edweek.org/ew/section/multimedia/map-coronavirus-and-school-closures.html>  <https://www.nytimes.com/interactive/2020/us/coronavirus-stay-at-home-order.html> |
| 11 | [https://governor.hawaii.gov/wp-content/uploads/2020/03/2003020-GOV-Emergency-Proclamation_COVID-19.pdf](https://governor.hawaii.gov/wp-content/uploads/2020/03/2003020-GOV-Emergency-Proclamation_COVID-19.pdfhttps://governor.hawaii.gov/newsroom/latest-news/office-of-the-governor-news-release-governor-ige-issues-statewide-order-to-stay-at-home-work-from-home-to-fight-covid-19/https://www.hawaiinewsnow.com/2020/03/18/live-governor-discuss-latest-efforts-stem-spread-coronavirus/)  [https://governor.hawaii.gov/newsroom/latest-news/office-of-the-governor-news-release-governor-ige-issues-statewide-order-to-stay-at-home-work-from-home-to-fight-covid-19/](https://governor.hawaii.gov/wp-content/uploads/2020/03/2003020-GOV-Emergency-Proclamation_COVID-19.pdfhttps://governor.hawaii.gov/newsroom/latest-news/office-of-the-governor-news-release-governor-ige-issues-statewide-order-to-stay-at-home-work-from-home-to-fight-covid-19/https://www.hawaiinewsnow.com/2020/03/18/live-governor-discuss-latest-efforts-stem-spread-coronavirus/)  [https://www.hawaiinewsnow.com/2020/03/18/live-governor-discuss-latest-efforts-stem-spread-coronavirus/](https://governor.hawaii.gov/wp-content/uploads/2020/03/2003020-GOV-Emergency-Proclamation_COVID-19.pdfhttps://governor.hawaii.gov/newsroom/latest-news/office-of-the-governor-news-release-governor-ige-issues-statewide-order-to-stay-at-home-work-from-home-to-fight-covid-19/https://www.hawaiinewsnow.com/2020/03/18/live-governor-discuss-latest-efforts-stem-spread-coronavirus/)  <https://ballotpedia.org/Travel_restrictions_issued_by_states_in_response_to_the_coronavirus_(COVID-19)_pandemic,_2020>  <https://www.edweek.org/ew/section/multimedia/map-coronavirus-and-school-closures.html>  <https://www.nytimes.com/interactive/2020/us/coronavirus-stay-at-home-order.html>  https://www.hawaiinewsnow.com/2020/04/20/under-new-rule-youre-now-required-wear-face-mask-most-public-settings-oahu/ |
| 12 | [https://coronavirus.idaho.gov/wp-content/uploads/sites/127/2020/03/proclamation_emergency-declaration_031320.pdf](https://coronavirus.idaho.gov/wp-content/uploads/sites/127/2020/03/proclamation_emergency-declaration_031320.pdfhttps://gov.idaho.gov/pressrelease/idaho-adopts-latest-federal-guidance-to-prevent-coronavirus-spread/)  [https://gov.idaho.gov/pressrelease/idaho-adopts-latest-federal-guidance-to-prevent-coronavirus-spread/](https://coronavirus.idaho.gov/wp-content/uploads/sites/127/2020/03/proclamation_emergency-declaration_031320.pdfhttps://gov.idaho.gov/pressrelease/idaho-adopts-latest-federal-guidance-to-prevent-coronavirus-spread/)  <https://ballotpedia.org/Travel_restrictions_issued_by_states_in_response_to_the_coronavirus_(COVID-19)_pandemic,_2020>  <https://www.edweek.org/ew/section/multimedia/map-coronavirus-and-school-closures.html>  <https://www.nytimes.com/interactive/2020/us/coronavirus-stay-at-home-order.html> |
| 13 | [https://www2.illinois.gov/Documents/ExecOrders/2020/ExecutiveOrder-2020-07.pdf](https://www2.illinois.gov/Documents/ExecOrders/2020/ExecutiveOrder-2020-07.pdfhttps://www2.illinois.gov/Documents/ExecOrders/2020/ExecutiveOrder-2020-10.pdf)  [https://www2.illinois.gov/Documents/ExecOrders/2020/ExecutiveOrder-2020-10.pdf](https://www2.illinois.gov/Documents/ExecOrders/2020/ExecutiveOrder-2020-07.pdfhttps://www2.illinois.gov/Documents/ExecOrders/2020/ExecutiveOrder-2020-10.pdf)  <https://www.edweek.org/ew/section/multimedia/map-coronavirus-and-school-closures.html>  <https://www.nytimes.com/interactive/2020/us/coronavirus-stay-at-home-order.html> |
| 14 | [https://www.in.gov/gov/files/20-02ExecutiveOrder(DeclarationofPublicHealthEmergencyforCOVID-19)FINAL.pdf](https://www.in.gov/gov/files/20-02ExecutiveOrder(DeclarationofPublicHealthEmergencyforCOVID-19)FINAL.pdfhttps://www.in.gov/gov/files/ExecutiveOrder20-04FurtherOrdersforPublicHealthEmergency.pdfhttps://www.in.gov/gov/files/Executive_Order_20-08_Stay_at_Home.pdf?mod=article_inlinehttps://en.wikipedia.org/wiki/2020_coronavirus_pandemic_in_Indiana)  [https://www.in.gov/gov/files/ExecutiveOrder20-04FurtherOrdersforPublicHealthEmergency.pdf](https://www.in.gov/gov/files/20-02ExecutiveOrder(DeclarationofPublicHealthEmergencyforCOVID-19)FINAL.pdfhttps://www.in.gov/gov/files/ExecutiveOrder20-04FurtherOrdersforPublicHealthEmergency.pdfhttps://www.in.gov/gov/files/Executive_Order_20-08_Stay_at_Home.pdf?mod=article_inlinehttps://en.wikipedia.org/wiki/2020_coronavirus_pandemic_in_Indiana)  [https://www.in.gov/gov/files/Executive_Order_20-08_Stay_at_Home.pdf?mod=article_inline](https://www.in.gov/gov/files/20-02ExecutiveOrder(DeclarationofPublicHealthEmergencyforCOVID-19)FINAL.pdfhttps://www.in.gov/gov/files/ExecutiveOrder20-04FurtherOrdersforPublicHealthEmergency.pdfhttps://www.in.gov/gov/files/Executive_Order_20-08_Stay_at_Home.pdf?mod=article_inlinehttps://en.wikipedia.org/wiki/2020_coronavirus_pandemic_in_Indiana)  [https://en.wikipedia.org/wiki/2020_coronavirus_pandemic_in_Indiana](https://www.in.gov/gov/files/20-02ExecutiveOrder(DeclarationofPublicHealthEmergencyforCOVID-19)FINAL.pdfhttps://www.in.gov/gov/files/ExecutiveOrder20-04FurtherOrdersforPublicHealthEmergency.pdfhttps://www.in.gov/gov/files/Executive_Order_20-08_Stay_at_Home.pdf?mod=article_inlinehttps://en.wikipedia.org/wiki/2020_coronavirus_pandemic_in_Indiana)  <https://www.edweek.org/ew/section/multimedia/map-coronavirus-and-school-closures.html>  <https://www.nytimes.com/interactive/2020/us/coronavirus-stay-at-home-order.html> |
| 15 | [https://governor.iowa.gov/press-release/gov-reynolds-signs-disaster-proclamation-following-additional-covid-19-cases-in-iowa](https://governor.iowa.gov/press-release/gov-reynolds-signs-disaster-proclamation-following-additional-covid-19-cases-in-iowahttps://governor.iowa.gov/press-release/gov-reynolds-recommends-iowa-schools-close-for-four-weeks-will-hold-a-press-0https://governor.iowa.gov/press-release/gov-reynolds-issues-a-state-of-public-health-disaster-emergencyhttps://governor.iowa.gov/press-release/gov-reynolds-issues-a-state-of-public-health-disaster-emergencyhttps://governor.iowa.gov/press-release/gov-reynolds-issues-a-state-of-public-health-disaster-emergency)  [https://governor.iowa.gov/press-release/gov-reynolds-recommends-iowa-schools-close-for-four-weeks-will-hold-a-press-0](https://governor.iowa.gov/press-release/gov-reynolds-signs-disaster-proclamation-following-additional-covid-19-cases-in-iowahttps://governor.iowa.gov/press-release/gov-reynolds-recommends-iowa-schools-close-for-four-weeks-will-hold-a-press-0https://governor.iowa.gov/press-release/gov-reynolds-issues-a-state-of-public-health-disaster-emergencyhttps://governor.iowa.gov/press-release/gov-reynolds-issues-a-state-of-public-health-disaster-emergencyhttps://governor.iowa.gov/press-release/gov-reynolds-issues-a-state-of-public-health-disaster-emergency)  [https://governor.iowa.gov/press-release/gov-reynolds-issues-a-state-of-public-health-disaster-emergency](https://governor.iowa.gov/press-release/gov-reynolds-signs-disaster-proclamation-following-additional-covid-19-cases-in-iowahttps://governor.iowa.gov/press-release/gov-reynolds-recommends-iowa-schools-close-for-four-weeks-will-hold-a-press-0https://governor.iowa.gov/press-release/gov-reynolds-issues-a-state-of-public-health-disaster-emergencyhttps://governor.iowa.gov/press-release/gov-reynolds-issues-a-state-of-public-health-disaster-emergencyhttps://governor.iowa.gov/press-release/gov-reynolds-issues-a-state-of-public-health-disaster-emergency)  [https://governor.iowa.gov/press-release/gov-reynolds-issues-a-state-of-public-health-disaster-emergency](https://governor.iowa.gov/press-release/gov-reynolds-signs-disaster-proclamation-following-additional-covid-19-cases-in-iowahttps://governor.iowa.gov/press-release/gov-reynolds-recommends-iowa-schools-close-for-four-weeks-will-hold-a-press-0https://governor.iowa.gov/press-release/gov-reynolds-issues-a-state-of-public-health-disaster-emergencyhttps://governor.iowa.gov/press-release/gov-reynolds-issues-a-state-of-public-health-disaster-emergencyhttps://governor.iowa.gov/press-release/gov-reynolds-issues-a-state-of-public-health-disaster-emergency)  [https://governor.iowa.gov/press-release/gov-reynolds-issues-a-state-of-public-health-disaster-emergency](https://governor.iowa.gov/press-release/gov-reynolds-signs-disaster-proclamation-following-additional-covid-19-cases-in-iowahttps://governor.iowa.gov/press-release/gov-reynolds-recommends-iowa-schools-close-for-four-weeks-will-hold-a-press-0https://governor.iowa.gov/press-release/gov-reynolds-issues-a-state-of-public-health-disaster-emergencyhttps://governor.iowa.gov/press-release/gov-reynolds-issues-a-state-of-public-health-disaster-emergencyhttps://governor.iowa.gov/press-release/gov-reynolds-issues-a-state-of-public-health-disaster-emergency)  <https://www.edweek.org/ew/section/multimedia/map-coronavirus-and-school-closures.html> |
| 16 | [https://governor.kansas.gov/wp-content/uploads/2020/03/20-03-Executed.pdf](https://governor.kansas.gov/wp-content/uploads/2020/03/20-03-Executed.pdfhttps://governor.kansas.gov/wp-content/uploads/2020/03/20-04-Executed.pdf)  [https://governor.kansas.gov/wp-content/uploads/2020/03/20-04-Executed.pdf](https://governor.kansas.gov/wp-content/uploads/2020/03/20-03-Executed.pdfhttps://governor.kansas.gov/wp-content/uploads/2020/03/20-04-Executed.pdf)  <https://ballotpedia.org/Travel_restrictions_issued_by_states_in_response_to_the_coronavirus_(COVID-19)_pandemic,_2020>  <https://www.edweek.org/ew/section/multimedia/map-coronavirus-and-school-closures.html>  <https://www.nytimes.com/interactive/2020/us/coronavirus-stay-at-home-order.html> |
| 17 | <https://governor.ky.gov/covid19>  <https://ballotpedia.org/Travel_restrictions_issued_by_states_in_response_to_the_coronavirus_(COVID-19)_pandemic,_2020>  <https://www.edweek.org/ew/section/multimedia/map-coronavirus-and-school-closures.html>  <https://www.nytimes.com/interactive/2020/us/coronavirus-stay-at-home-order.html> |
| 18 | [https://gov.louisiana.gov/index.cfm/newsroom/detail/2400](https://gov.louisiana.gov/index.cfm/newsroom/detail/2400https://gov.louisiana.gov/assets/Proclamations/2020/JBE-33-2020.pdf)  [https://gov.louisiana.gov/assets/Proclamations/2020/JBE-33-2020.pdf](https://gov.louisiana.gov/index.cfm/newsroom/detail/2400https://gov.louisiana.gov/assets/Proclamations/2020/JBE-33-2020.pdf)  <https://www.edweek.org/ew/section/multimedia/map-coronavirus-and-school-closures.html>  <https://www.nytimes.com/interactive/2020/us/coronavirus-stay-at-home-order.html> |
| 19 | [https://www.maine.gov/governor/mills/sites/maine.gov.governor.mills/files/inline-files/Proclamation%20of%20State%20of%20Civil%20Emergency%20To%20Further%20Protect%20Public%20Health.pdf](https://www.maine.gov/governor/mills/sites/maine.gov.governor.mills/files/inline-files/Proclamation%20of%20State%20of%20Civil%20Emergency%20To%20Further%20Protect%20Public%20Health.pdfhttps://www.maine.gov/governor/mills/sites/maine.gov.governor.mills/files/inline-files/EO%2014%20An%20Order%20to%20Protect%20Public%20Health.pdfhttps://www.wagmtv.com/content/news/All-non-essential-businesses-ordered-to-close-569063571.html)  [https://www.maine.gov/governor/mills/sites/maine.gov.governor.mills/files/inline-files/EO%2014%20An%20Order%20to%20Protect%20Public%20Health.pdf](https://www.maine.gov/governor/mills/sites/maine.gov.governor.mills/files/inline-files/Proclamation%20of%20State%20of%20Civil%20Emergency%20To%20Further%20Protect%20Public%20Health.pdfhttps://www.maine.gov/governor/mills/sites/maine.gov.governor.mills/files/inline-files/EO%2014%20An%20Order%20to%20Protect%20Public%20Health.pdfhttps://www.wagmtv.com/content/news/All-non-essential-businesses-ordered-to-close-569063571.html)  [https://www.wagmtv.com/content/news/All-non-essential-businesses-ordered-to-close-569063571.html](https://www.maine.gov/governor/mills/sites/maine.gov.governor.mills/files/inline-files/Proclamation%20of%20State%20of%20Civil%20Emergency%20To%20Further%20Protect%20Public%20Health.pdfhttps://www.maine.gov/governor/mills/sites/maine.gov.governor.mills/files/inline-files/EO%2014%20An%20Order%20to%20Protect%20Public%20Health.pdfhttps://www.wagmtv.com/content/news/All-non-essential-businesses-ordered-to-close-569063571.html)  <https://ballotpedia.org/Travel_restrictions_issued_by_states_in_response_to_the_coronavirus_(COVID-19)_pandemic,_2020>  <https://www.edweek.org/ew/section/multimedia/map-coronavirus-and-school-closures.html>  <https://www.nytimes.com/interactive/2020/us/coronavirus-stay-at-home-order.html> |
| 20 | [https://governor.maryland.gov/wp-content/uploads/2020/03/Proclamation-COVID-19.pdf](https://governor.maryland.gov/wp-content/uploads/2020/03/Proclamation-COVID-19.pdfhttps://governor.maryland.gov/wp-content/uploads/2020/03/Executive-Order-Amending-Large-Gatherings.pdfhttps://governor.maryland.gov/2020/03/23/governor-hogan-announces-closure-of-all-non-essential-businesses-175-million-relief-package-for-workers-and-small-businesses-affected-by-covid-19/)  [https://governor.maryland.gov/wp-content/uploads/2020/03/Executive-Order-Amending-Large-Gatherings.pdf](https://governor.maryland.gov/wp-content/uploads/2020/03/Proclamation-COVID-19.pdfhttps://governor.maryland.gov/wp-content/uploads/2020/03/Executive-Order-Amending-Large-Gatherings.pdfhttps://governor.maryland.gov/2020/03/23/governor-hogan-announces-closure-of-all-non-essential-businesses-175-million-relief-package-for-workers-and-small-businesses-affected-by-covid-19/)  [https://governor.maryland.gov/2020/03/23/governor-hogan-announces-closure-of-all-non-essential-businesses-175-million-relief-package-for-workers-and-small-businesses-affected-by-covid-19/](https://governor.maryland.gov/wp-content/uploads/2020/03/Proclamation-COVID-19.pdfhttps://governor.maryland.gov/wp-content/uploads/2020/03/Executive-Order-Amending-Large-Gatherings.pdfhttps://governor.maryland.gov/2020/03/23/governor-hogan-announces-closure-of-all-non-essential-businesses-175-million-relief-package-for-workers-and-small-businesses-affected-by-covid-19/)  <https://ballotpedia.org/Travel_restrictions_issued_by_states_in_response_to_the_coronavirus_(COVID-19)_pandemic,_2020>  <https://www.edweek.org/ew/section/multimedia/map-coronavirus-and-school-closures.html>  <https://www.nytimes.com/interactive/2020/us/coronavirus-stay-at-home-order.html> |
| 21 | [https://www.mass.gov/info-details/covid-19-state-of-emergency](https://www.mass.gov/info-details/covid-19-state-of-emergencyhttps://www.thebostoncalendar.com/events/massachusetts-bans-dining-at-restaurants-bars-closes-all-schools-and-forbids-gatherings-over-25https://www.mass.gov/doc/march-23-2020-essential-services-and-revised-gatherings-order/download)  [https://www.thebostoncalendar.com/events/massachusetts-bans-dining-at-restaurants-bars-closes-all-schools-and-forbids-gatherings-over-25](https://www.mass.gov/info-details/covid-19-state-of-emergencyhttps://www.thebostoncalendar.com/events/massachusetts-bans-dining-at-restaurants-bars-closes-all-schools-and-forbids-gatherings-over-25https://www.mass.gov/doc/march-23-2020-essential-services-and-revised-gatherings-order/download)  [https://www.mass.gov/doc/march-23-2020-essential-services-and-revised-gatherings-order/download](https://www.mass.gov/info-details/covid-19-state-of-emergencyhttps://www.thebostoncalendar.com/events/massachusetts-bans-dining-at-restaurants-bars-closes-all-schools-and-forbids-gatherings-over-25https://www.mass.gov/doc/march-23-2020-essential-services-and-revised-gatherings-order/download)  <https://ballotpedia.org/Travel_restrictions_issued_by_states_in_response_to_the_coronavirus_(COVID-19)_pandemic,_2020>  <https://www.edweek.org/ew/section/multimedia/map-coronavirus-and-school-closures.html>  <https://www.nytimes.com/interactive/2020/us/coronavirus-stay-at-home-order.html>  https://www.baltimoresun.com/coronavirus/bs-md-make-a-mask-20200415-hzmnoed2pndq7abfitbfk42lh4-story.html |
| 22 | [https://www.michigan.gov/whitmer/0,9309,7-387-90499_90705-521576--,00.html](https://www.michigan.gov/whitmer/0,9309,7-387-90499_90705-521576--,00.htmlhttps://www.michigan.gov/whitmer/0,9309,7-387-90499_90705-521595--,00.htmlhttps://www.michigan.gov/whitmer/0,9309,7-387-90499_90705-521789--,00.html)  [https://www.michigan.gov/whitmer/0,9309,7-387-90499_90705-521595--,00.html](https://www.michigan.gov/whitmer/0,9309,7-387-90499_90705-521576--,00.htmlhttps://www.michigan.gov/whitmer/0,9309,7-387-90499_90705-521595--,00.htmlhttps://www.michigan.gov/whitmer/0,9309,7-387-90499_90705-521789--,00.html)  [https://www.michigan.gov/whitmer/0,9309,7-387-90499_90705-521789--,00.html](https://www.michigan.gov/whitmer/0,9309,7-387-90499_90705-521576--,00.htmlhttps://www.michigan.gov/whitmer/0,9309,7-387-90499_90705-521595--,00.htmlhttps://www.michigan.gov/whitmer/0,9309,7-387-90499_90705-521789--,00.html)  <https://www.edweek.org/ew/section/multimedia/map-coronavirus-and-school-closures.html>  <https://www.nytimes.com/interactive/2020/us/coronavirus-stay-at-home-order.html> |
| 23 | <https://www.leg.state.mn.us/lrl/execorders/eoresults>  <https://www.edweek.org/ew/section/multimedia/map-coronavirus-and-school-closures.html>  <https://www.nytimes.com/interactive/2020/us/coronavirus-stay-at-home-order.html> |
| 24 | [https://www.sos.ms.gov/Content/documents/about_us/WhatsNew/GovernorProclomationPublicHealth.pdf](https://www.sos.ms.gov/Content/documents/about_us/WhatsNew/GovernorProclomationPublicHealth.pdfhttps://www.sos.ms.gov/Content/documents/ed_pubs/Exec%20Orders/1463.pdfhttps://www.wlox.com/2020/03/25/list-all-businesses-operations-that-are-considered-essential-mississippi/)  [https://www.sos.ms.gov/Content/documents/ed_pubs/Exec%20Orders/1463.pdf](https://www.sos.ms.gov/Content/documents/about_us/WhatsNew/GovernorProclomationPublicHealth.pdfhttps://www.sos.ms.gov/Content/documents/ed_pubs/Exec%20Orders/1463.pdfhttps://www.wlox.com/2020/03/25/list-all-businesses-operations-that-are-considered-essential-mississippi/)  [https://www.wlox.com/2020/03/25/list-all-businesses-operations-that-are-considered-essential-mississippi/](https://www.sos.ms.gov/Content/documents/about_us/WhatsNew/GovernorProclomationPublicHealth.pdfhttps://www.sos.ms.gov/Content/documents/ed_pubs/Exec%20Orders/1463.pdfhttps://www.wlox.com/2020/03/25/list-all-businesses-operations-that-are-considered-essential-mississippi/)  <https://www.edweek.org/ew/section/multimedia/map-coronavirus-and-school-closures.html>  <https://www.nytimes.com/interactive/2020/us/coronavirus-stay-at-home-order.html> |
| 25 | [https://governor.mo.gov/press-releases/archive/30-day-recap-missouris-covid-19-response-efforts](https://governor.mo.gov/press-releases/archive/30-day-recap-missouris-covid-19-response-effortshttps://www.lakeexpo.com/news/coronavirus/missouri-governor-orders-closure-of-restaurant-dining-rooms-bans-gatherings-of-more-than-10/article_8f425620-6d27-11ea-afb8-370db8589bc0.html)  [https://www.lakeexpo.com/news/coronavirus/missouri-governor-orders-closure-of-restaurant-dining-rooms-bans-gatherings-of-more-than-10/article_8f425620-6d27-11ea-afb8-370db8589bc0.html](https://governor.mo.gov/press-releases/archive/30-day-recap-missouris-covid-19-response-effortshttps://www.lakeexpo.com/news/coronavirus/missouri-governor-orders-closure-of-restaurant-dining-rooms-bans-gatherings-of-more-than-10/article_8f425620-6d27-11ea-afb8-370db8589bc0.html)  <https://www.edweek.org/ew/section/multimedia/map-coronavirus-and-school-closures.html>  <https://www.nytimes.com/interactive/2020/us/coronavirus-stay-at-home-order.html> |
| 26 | [http://governor.mt.gov/Pressroom/governor-bullock-declares-state-of-emergency-in-montana-related-to-covid-19](http://governor.mt.gov/Pressroom/governor-bullock-declares-state-of-emergency-in-montana-related-to-covid-19http://governor.mt.gov/Pressroom/governor-bullock-directs-the-closure-of-public-k-12-schools-for-two-weeks-strongly-recommends-social-distancing-measures-to-slow-the-spread-of-covid-19http://governor.mt.gov/Pressroom/governor-bullock-declares-state-of-emergency-in-montana-related-to-covid-19http://governor.mt.gov/Pressroom/governor-bullock-declares-state-of-emergency-in-montana-related-to-covid-19)  [http://governor.mt.gov/Pressroom/governor-bullock-directs-the-closure-of-public-k-12-schools-for-two-weeks-strongly-recommends-social-distancing-measures-to-slow-the-spread-of-covid-19](http://governor.mt.gov/Pressroom/governor-bullock-declares-state-of-emergency-in-montana-related-to-covid-19http://governor.mt.gov/Pressroom/governor-bullock-directs-the-closure-of-public-k-12-schools-for-two-weeks-strongly-recommends-social-distancing-measures-to-slow-the-spread-of-covid-19http://governor.mt.gov/Pressroom/governor-bullock-declares-state-of-emergency-in-montana-related-to-covid-19http://governor.mt.gov/Pressroom/governor-bullock-declares-state-of-emergency-in-montana-related-to-covid-19)  [http://governor.mt.gov/Pressroom/governor-bullock-declares-state-of-emergency-in-montana-related-to-covid-19](http://governor.mt.gov/Pressroom/governor-bullock-declares-state-of-emergency-in-montana-related-to-covid-19http://governor.mt.gov/Pressroom/governor-bullock-directs-the-closure-of-public-k-12-schools-for-two-weeks-strongly-recommends-social-distancing-measures-to-slow-the-spread-of-covid-19http://governor.mt.gov/Pressroom/governor-bullock-declares-state-of-emergency-in-montana-related-to-covid-19http://governor.mt.gov/Pressroom/governor-bullock-declares-state-of-emergency-in-montana-related-to-covid-19)  [http://governor.mt.gov/Pressroom/governor-bullock-declares-state-of-emergency-in-montana-related-to-covid-19](http://governor.mt.gov/Pressroom/governor-bullock-declares-state-of-emergency-in-montana-related-to-covid-19http://governor.mt.gov/Pressroom/governor-bullock-directs-the-closure-of-public-k-12-schools-for-two-weeks-strongly-recommends-social-distancing-measures-to-slow-the-spread-of-covid-19http://governor.mt.gov/Pressroom/governor-bullock-declares-state-of-emergency-in-montana-related-to-covid-19http://governor.mt.gov/Pressroom/governor-bullock-declares-state-of-emergency-in-montana-related-to-covid-19)  <https://ballotpedia.org/Travel_restrictions_issued_by_states_in_response_to_the_coronavirus_(COVID-19)_pandemic,_2020>  <https://www.edweek.org/ew/section/multimedia/map-coronavirus-and-school-closures.html>  <https://www.nytimes.com/interactive/2020/us/coronavirus-stay-at-home-order.html> |
| 27 | [https://governor.nebraska.gov/press/gov-ricketts-issues-emergency-declaration-covid-19](https://governor.nebraska.gov/press/gov-ricketts-issues-emergency-declaration-covid-19https://governor.nebraska.gov/press/gov-ricketts-further-limits-events-gatherings-prevent-covid-19-spreadhttps://governor.nebraska.gov/press/gov-ricketts-reminds-nebraskans-cdc-guidance-limiting-gatherings-ten-peoplehttps://governor.nebraska.gov/press/gov-ricketts-announces-directed-health-measure-remaining-counties-asks-nebraskans-furtherhttps://governor.nebraska.gov/press/gov-ricketts-announces-directed-health-measure-remaining-counties-asks-nebraskans-furtherhttps://governor.nebraska.gov/press/gov-ricketts-adds-additional-businesses-state%E2%80%99s-directed-health-measure)  [https://governor.nebraska.gov/press/gov-ricketts-further-limits-events-gatherings-prevent-covid-19-spread](https://governor.nebraska.gov/press/gov-ricketts-issues-emergency-declaration-covid-19https://governor.nebraska.gov/press/gov-ricketts-further-limits-events-gatherings-prevent-covid-19-spreadhttps://governor.nebraska.gov/press/gov-ricketts-reminds-nebraskans-cdc-guidance-limiting-gatherings-ten-peoplehttps://governor.nebraska.gov/press/gov-ricketts-announces-directed-health-measure-remaining-counties-asks-nebraskans-furtherhttps://governor.nebraska.gov/press/gov-ricketts-announces-directed-health-measure-remaining-counties-asks-nebraskans-furtherhttps://governor.nebraska.gov/press/gov-ricketts-adds-additional-businesses-state%E2%80%99s-directed-health-measure)  [https://governor.nebraska.gov/press/gov-ricketts-reminds-nebraskans-cdc-guidance-limiting-gatherings-ten-people](https://governor.nebraska.gov/press/gov-ricketts-issues-emergency-declaration-covid-19https://governor.nebraska.gov/press/gov-ricketts-further-limits-events-gatherings-prevent-covid-19-spreadhttps://governor.nebraska.gov/press/gov-ricketts-reminds-nebraskans-cdc-guidance-limiting-gatherings-ten-peoplehttps://governor.nebraska.gov/press/gov-ricketts-announces-directed-health-measure-remaining-counties-asks-nebraskans-furtherhttps://governor.nebraska.gov/press/gov-ricketts-announces-directed-health-measure-remaining-counties-asks-nebraskans-furtherhttps://governor.nebraska.gov/press/gov-ricketts-adds-additional-businesses-state%E2%80%99s-directed-health-measure)  [https://governor.nebraska.gov/press/gov-ricketts-announces-directed-health-measure-remaining-counties-asks-nebraskans-further](https://governor.nebraska.gov/press/gov-ricketts-issues-emergency-declaration-covid-19https://governor.nebraska.gov/press/gov-ricketts-further-limits-events-gatherings-prevent-covid-19-spreadhttps://governor.nebraska.gov/press/gov-ricketts-reminds-nebraskans-cdc-guidance-limiting-gatherings-ten-peoplehttps://governor.nebraska.gov/press/gov-ricketts-announces-directed-health-measure-remaining-counties-asks-nebraskans-furtherhttps://governor.nebraska.gov/press/gov-ricketts-announces-directed-health-measure-remaining-counties-asks-nebraskans-furtherhttps://governor.nebraska.gov/press/gov-ricketts-adds-additional-businesses-state%E2%80%99s-directed-health-measure)  [https://governor.nebraska.gov/press/gov-ricketts-announces-directed-health-measure-remaining-counties-asks-nebraskans-further](https://governor.nebraska.gov/press/gov-ricketts-issues-emergency-declaration-covid-19https://governor.nebraska.gov/press/gov-ricketts-further-limits-events-gatherings-prevent-covid-19-spreadhttps://governor.nebraska.gov/press/gov-ricketts-reminds-nebraskans-cdc-guidance-limiting-gatherings-ten-peoplehttps://governor.nebraska.gov/press/gov-ricketts-announces-directed-health-measure-remaining-counties-asks-nebraskans-furtherhttps://governor.nebraska.gov/press/gov-ricketts-announces-directed-health-measure-remaining-counties-asks-nebraskans-furtherhttps://governor.nebraska.gov/press/gov-ricketts-adds-additional-businesses-state%E2%80%99s-directed-health-measure)  [https://governor.nebraska.gov/press/gov-ricketts-adds-additional-businesses-state%E2%80%99s-directed-health-measure](https://governor.nebraska.gov/press/gov-ricketts-issues-emergency-declaration-covid-19https://governor.nebraska.gov/press/gov-ricketts-further-limits-events-gatherings-prevent-covid-19-spreadhttps://governor.nebraska.gov/press/gov-ricketts-reminds-nebraskans-cdc-guidance-limiting-gatherings-ten-peoplehttps://governor.nebraska.gov/press/gov-ricketts-announces-directed-health-measure-remaining-counties-asks-nebraskans-furtherhttps://governor.nebraska.gov/press/gov-ricketts-announces-directed-health-measure-remaining-counties-asks-nebraskans-furtherhttps://governor.nebraska.gov/press/gov-ricketts-adds-additional-businesses-state%E2%80%99s-directed-health-measure)  <https://ballotpedia.org/Travel_restrictions_issued_by_states_in_response_to_the_coronavirus_(COVID-19)_pandemic,_2020>  <https://www.edweek.org/ew/section/multimedia/map-coronavirus-and-school-closures.html>  <https://www.nytimes.com/interactive/2020/us/coronavirus-stay-at-home-order.html> |
| 28 | [http://gov.nv.gov/News/Press/2020/Governor_Sisolak_Declares_State_of_Emergency_in_Response_to_COVID-19/](http://gov.nv.gov/News/Press/2020/Governor_Sisolak_Declares_State_of_Emergency_in_Response_to_COVID-19/http://gov.nv.gov/News/Emergency_Orders/2020/2020-03-24_-_COVID-19_Declaration_of_Emergency_Directive_007/http://gov.nv.gov/News/Press/2020/Governor_Sisolak_Announces_COVID-19_Risk_Mitigation_Initiatives/http://gov.nv.gov/News/Press/2020/Governor_Sisolak_Announces_COVID-19_Risk_Mitigation_Initiatives/)  [http://gov.nv.gov/News/Emergency_Orders/2020/2020-03-24_-_COVID-19_Declaration_of_Emergency_Directive_007/](http://gov.nv.gov/News/Press/2020/Governor_Sisolak_Declares_State_of_Emergency_in_Response_to_COVID-19/http://gov.nv.gov/News/Emergency_Orders/2020/2020-03-24_-_COVID-19_Declaration_of_Emergency_Directive_007/http://gov.nv.gov/News/Press/2020/Governor_Sisolak_Announces_COVID-19_Risk_Mitigation_Initiatives/http://gov.nv.gov/News/Press/2020/Governor_Sisolak_Announces_COVID-19_Risk_Mitigation_Initiatives/)  [http://gov.nv.gov/News/Press/2020/Governor_Sisolak_Announces_COVID-19_Risk_Mitigation_Initiatives/](http://gov.nv.gov/News/Press/2020/Governor_Sisolak_Declares_State_of_Emergency_in_Response_to_COVID-19/http://gov.nv.gov/News/Emergency_Orders/2020/2020-03-24_-_COVID-19_Declaration_of_Emergency_Directive_007/http://gov.nv.gov/News/Press/2020/Governor_Sisolak_Announces_COVID-19_Risk_Mitigation_Initiatives/http://gov.nv.gov/News/Press/2020/Governor_Sisolak_Announces_COVID-19_Risk_Mitigation_Initiatives/)  [http://gov.nv.gov/News/Press/2020/Governor_Sisolak_Announces_COVID-19_Risk_Mitigation_Initiatives/](http://gov.nv.gov/News/Press/2020/Governor_Sisolak_Declares_State_of_Emergency_in_Response_to_COVID-19/http://gov.nv.gov/News/Emergency_Orders/2020/2020-03-24_-_COVID-19_Declaration_of_Emergency_Directive_007/http://gov.nv.gov/News/Press/2020/Governor_Sisolak_Announces_COVID-19_Risk_Mitigation_Initiatives/http://gov.nv.gov/News/Press/2020/Governor_Sisolak_Announces_COVID-19_Risk_Mitigation_Initiatives/)  <https://www.edweek.org/ew/section/multimedia/map-coronavirus-and-school-closures.html>  <https://www.nytimes.com/interactive/2020/us/coronavirus-stay-at-home-order.html> |
| 29 | <https://www.governor.nh.gov/news-media/emergency-orders/documents/emergency-order-2.pdf>  <https://www.governor.nh.gov/news-media/emergency-orders/documents/emergency-order-2.pdf>  <https://www.governor.nh.gov/news-media/emergency-orders/documents/emergency-order-16.pdf>  <https://www.governor.nh.gov/news-media/press-2020/20200316-covid-10-businesses.htm>  <https://www.governor.nh.gov/news-media/press-2020/20200326-emergency-order-17.htm>  <https://ballotpedia.org/Travel_restrictions_issued_by_states_in_response_to_the_coronavirus_(COVID-19)_pandemic,_2020>  <https://www.edweek.org/ew/section/multimedia/map-coronavirus-and-school-closures.html>  <https://www.nytimes.com/interactive/2020/us/coronavirus-stay-at-home-order.html> |
| 30 | <https://nj.gov/governor/news/news/562020/approved/20200309b.shtml>  <https://nj.gov/governor/news/news/562020/approved/20200316c.shtml>  <https://nj.gov/governor/news/news/562020/approved/20200316c.shtml>  <https://nj.gov/governor/news/news/562020/approved/20200317a.shtml>  <https://www.edweek.org/ew/section/multimedia/map-coronavirus-and-school-closures.html>  <https://www.nytimes.com/interactive/2020/us/coronavirus-stay-at-home-order.html>  <https://www.phillyvoice.com/new-jersey-face-masks-required-indoor-activities-coronavirus-covid-19/> |
| 31 | <https://www.governor.state.nm.us/wp-content/uploads/2020/03/Executive-Order-2020-004.pdf>  <https://www.governor.state.nm.us/2020/03/12/health-secretary-issues-public-health-order-suspending-mass-gatherings-in-new-mexico/>  <https://www.governor.state.nm.us/2020/03/15/health-secretary-to-amend-public-health-order-adding-new-restrictions-to-public-gatherings/>  <https://www.governor.state.nm.us/2020/03/18/new-mexico-to-order-additional-closures-to-limit-spread-of-covid-19/>  <https://www.governor.state.nm.us/2020/03/18/new-mexico-to-order-additional-closures-to-limit-spread-of-covid-19/>  <https://www.governor.state.nm.us/2020/03/18/new-mexico-to-order-additional-closures-to-limit-spread-of-covid-19/>  <https://ballotpedia.org/Travel_restrictions_issued_by_states_in_response_to_the_coronavirus_(COVID-19)_pandemic,_2020>  <https://www.edweek.org/ew/section/multimedia/map-coronavirus-and-school-closures.html>  <https://www.nytimes.com/interactive/2020/us/coronavirus-stay-at-home-order.html> |
| 32 | [https://www.governor.ny.gov/news/no-202-declaring-disaster-emergency-state-new-york](https://www.governor.ny.gov/news/no-202-declaring-disaster-emergency-state-new-yorkhttps://www.governor.ny.gov/news/no-2021-continuing-temporary-suspension-and-modification-laws-relating-disaster-emergencyhttps://www.governor.ny.gov/news/no-2023-continuing-temporary-suspension-and-modification-laws-relating-disaster-emergencyhttps://www.governor.ny.gov/news/no-2023-continuing-temporary-suspension-and-modification-laws-relating-disaster-emergencyhttps://www.governor.ny.gov/news/no-2023-continuing-temporary-suspension-and-modification-laws-relating-disaster-emergency)  [https://www.governor.ny.gov/news/no-2021-continuing-temporary-suspension-and-modification-laws-relating-disaster-emergency](https://www.governor.ny.gov/news/no-202-declaring-disaster-emergency-state-new-yorkhttps://www.governor.ny.gov/news/no-2021-continuing-temporary-suspension-and-modification-laws-relating-disaster-emergencyhttps://www.governor.ny.gov/news/no-2023-continuing-temporary-suspension-and-modification-laws-relating-disaster-emergencyhttps://www.governor.ny.gov/news/no-2023-continuing-temporary-suspension-and-modification-laws-relating-disaster-emergencyhttps://www.governor.ny.gov/news/no-2023-continuing-temporary-suspension-and-modification-laws-relating-disaster-emergency)  [https://www.governor.ny.gov/news/no-2023-continuing-temporary-suspension-and-modification-laws-relating-disaster-emergency](https://www.governor.ny.gov/news/no-202-declaring-disaster-emergency-state-new-yorkhttps://www.governor.ny.gov/news/no-2021-continuing-temporary-suspension-and-modification-laws-relating-disaster-emergencyhttps://www.governor.ny.gov/news/no-2023-continuing-temporary-suspension-and-modification-laws-relating-disaster-emergencyhttps://www.governor.ny.gov/news/no-2023-continuing-temporary-suspension-and-modification-laws-relating-disaster-emergencyhttps://www.governor.ny.gov/news/no-2023-continuing-temporary-suspension-and-modification-laws-relating-disaster-emergency)  [https://www.governor.ny.gov/news/no-2023-continuing-temporary-suspension-and-modification-laws-relating-disaster-emergency](https://www.governor.ny.gov/news/no-202-declaring-disaster-emergency-state-new-yorkhttps://www.governor.ny.gov/news/no-2021-continuing-temporary-suspension-and-modification-laws-relating-disaster-emergencyhttps://www.governor.ny.gov/news/no-2023-continuing-temporary-suspension-and-modification-laws-relating-disaster-emergencyhttps://www.governor.ny.gov/news/no-2023-continuing-temporary-suspension-and-modification-laws-relating-disaster-emergencyhttps://www.governor.ny.gov/news/no-2023-continuing-temporary-suspension-and-modification-laws-relating-disaster-emergency)  [https://www.governor.ny.gov/news/no-2023-continuing-temporary-suspension-and-modification-laws-relating-disaster-emergency](https://www.governor.ny.gov/news/no-202-declaring-disaster-emergency-state-new-yorkhttps://www.governor.ny.gov/news/no-2021-continuing-temporary-suspension-and-modification-laws-relating-disaster-emergencyhttps://www.governor.ny.gov/news/no-2023-continuing-temporary-suspension-and-modification-laws-relating-disaster-emergencyhttps://www.governor.ny.gov/news/no-2023-continuing-temporary-suspension-and-modification-laws-relating-disaster-emergencyhttps://www.governor.ny.gov/news/no-2023-continuing-temporary-suspension-and-modification-laws-relating-disaster-emergency)  <https://www.edweek.org/ew/section/multimedia/map-coronavirus-and-school-closures.html>  <https://www.nytimes.com/interactive/2020/us/coronavirus-stay-at-home-order.html>  <https://thehill.com/policy/healthcare/492967-new-york-to-require-all-people-to-wear-masks-when-in-public> |
| 33 | <https://governor.nc.gov/news/governor-cooper-declares-state-emergency-respond-coronavirus-covid-19>  <https://governor.nc.gov/news/governor-cooper-issues-executive-order-closing-k-12-public-schools-and-banning-gatherings-more>  <https://governor.nc.gov/news/governor-cooper-extends-school-closure-date-may-orders-group-limit-50-people>  <https://governor.nc.gov/news/north-carolina-close-restaurants-and-bars-dine-customers-allow-takeout-and-delivery-operations>  <https://www.edweek.org/ew/section/multimedia/map-coronavirus-and-school-closures.html>  <https://www.nytimes.com/interactive/2020/us/coronavirus-stay-at-home-order.html> |
| 34 | <https://www.governor.nd.gov/sites/www/files/documents/EO%202020-03.pdf>  <https://www.governor.nd.gov/news/burgum-orders-bars-restaurants-closed-site-patrons-provides-additional-guidance-k-12-schools>  <https://www.governor.nd.gov/news/burgum-orders-bars-restaurants-closed-site-patrons-provides-additional-guidance-k-12-schools>  <https://www.governor.nd.gov/news/burgum-expands-business-closures-protect-health-suspends-unemployment-taxes-help-employers>  <https://ballotpedia.org/Travel_restrictions_issued_by_states_in_response_to_the_coronavirus_(COVID-19)_pandemic,_2020>  <https://www.edweek.org/ew/section/multimedia/map-coronavirus-and-school-closures.html> |
| 35 | [https://coronavirus.ohio.gov/wps/portal/gov/covid-19/resources/news-releases-news-you-can-use/gov-dewine-signs-emergency-order-regarding-coronavirus-response](https://coronavirus.ohio.gov/wps/portal/gov/covid-19/resources/news-releases-news-you-can-use/gov-dewine-signs-emergency-order-regarding-coronavirus-responsehttps://coronavirus.ohio.gov/wps/portal/gov/covid-19/resources/news-releases-news-you-can-use/ohio-bans-mass-gatherings-of-100-or-morehttps://coronavirus.ohio.gov/wps/portal/gov/covid-19/resources/news-releases-news-you-can-use/governor-dewine-orders-ohio-bars-restaurants-to-close)  [https://coronavirus.ohio.gov/wps/portal/gov/covid-19/resources/news-releases-news-you-can-use/ohio-bans-mass-gatherings-of-100-or-more](https://coronavirus.ohio.gov/wps/portal/gov/covid-19/resources/news-releases-news-you-can-use/gov-dewine-signs-emergency-order-regarding-coronavirus-responsehttps://coronavirus.ohio.gov/wps/portal/gov/covid-19/resources/news-releases-news-you-can-use/ohio-bans-mass-gatherings-of-100-or-morehttps://coronavirus.ohio.gov/wps/portal/gov/covid-19/resources/news-releases-news-you-can-use/governor-dewine-orders-ohio-bars-restaurants-to-close)  [https://coronavirus.ohio.gov/wps/portal/gov/covid-19/resources/news-releases-news-you-can-use/governor-dewine-orders-ohio-bars-restaurants-to-close](https://coronavirus.ohio.gov/wps/portal/gov/covid-19/resources/news-releases-news-you-can-use/gov-dewine-signs-emergency-order-regarding-coronavirus-responsehttps://coronavirus.ohio.gov/wps/portal/gov/covid-19/resources/news-releases-news-you-can-use/ohio-bans-mass-gatherings-of-100-or-morehttps://coronavirus.ohio.gov/wps/portal/gov/covid-19/resources/news-releases-news-you-can-use/governor-dewine-orders-ohio-bars-restaurants-to-close)  <https://www.edweek.org/ew/section/multimedia/map-coronavirus-and-school-closures.html>  <https://www.nytimes.com/interactive/2020/us/coronavirus-stay-at-home-order.html> |
| 36 | [https://www.sos.ok.gov/documents/executive/1913.pdf](https://www.sos.ok.gov/documents/executive/1913.pdfhttps://www.sos.ok.gov/documents/executive/1919.pdfhttps://www.sos.ok.gov/documents/executive/1919.pdf)  [https://www.sos.ok.gov/documents/executive/1919.pdf](https://www.sos.ok.gov/documents/executive/1913.pdfhttps://www.sos.ok.gov/documents/executive/1919.pdfhttps://www.sos.ok.gov/documents/executive/1919.pdf)  [https://www.sos.ok.gov/documents/executive/1919.pdf](https://www.sos.ok.gov/documents/executive/1913.pdfhttps://www.sos.ok.gov/documents/executive/1919.pdfhttps://www.sos.ok.gov/documents/executive/1919.pdf)  <https://ballotpedia.org/Travel_restrictions_issued_by_states_in_response_to_the_coronavirus_(COVID-19)_pandemic,_2020>  <https://www.edweek.org/ew/section/multimedia/map-coronavirus-and-school-closures.html> |
| 37 | [https://www.oregon.gov/gov/Documents/executive_orders/eo_20-03.pdf](https://www.oregon.gov/gov/Documents/executive_orders/eo_20-03.pdfhttps://www.oregon.gov/gov/Documents/executive_orders/eo_20-05.pdfhttps://www.oregon.gov/gov/Documents/executive_orders/eo_20-07.pdfhttps://www.oregon.gov/gov/Documents/executive_orders/eo_20-07.pdfhttps://www.oregon.gov/gov/Documents/executive_orders/eo_20-12.pdf)  [https://www.oregon.gov/gov/Documents/executive_orders/eo_20-05.pdf](https://www.oregon.gov/gov/Documents/executive_orders/eo_20-03.pdfhttps://www.oregon.gov/gov/Documents/executive_orders/eo_20-05.pdfhttps://www.oregon.gov/gov/Documents/executive_orders/eo_20-07.pdfhttps://www.oregon.gov/gov/Documents/executive_orders/eo_20-07.pdfhttps://www.oregon.gov/gov/Documents/executive_orders/eo_20-12.pdf)  [https://www.oregon.gov/gov/Documents/executive_orders/eo_20-07.pdf](https://www.oregon.gov/gov/Documents/executive_orders/eo_20-03.pdfhttps://www.oregon.gov/gov/Documents/executive_orders/eo_20-05.pdfhttps://www.oregon.gov/gov/Documents/executive_orders/eo_20-07.pdfhttps://www.oregon.gov/gov/Documents/executive_orders/eo_20-07.pdfhttps://www.oregon.gov/gov/Documents/executive_orders/eo_20-12.pdf)  [https://www.oregon.gov/gov/Documents/executive_orders/eo_20-07.pdf](https://www.oregon.gov/gov/Documents/executive_orders/eo_20-03.pdfhttps://www.oregon.gov/gov/Documents/executive_orders/eo_20-05.pdfhttps://www.oregon.gov/gov/Documents/executive_orders/eo_20-07.pdfhttps://www.oregon.gov/gov/Documents/executive_orders/eo_20-07.pdfhttps://www.oregon.gov/gov/Documents/executive_orders/eo_20-12.pdf)  [https://www.oregon.gov/gov/Documents/executive_orders/eo_20-12.pdf](https://www.oregon.gov/gov/Documents/executive_orders/eo_20-03.pdfhttps://www.oregon.gov/gov/Documents/executive_orders/eo_20-05.pdfhttps://www.oregon.gov/gov/Documents/executive_orders/eo_20-07.pdfhttps://www.oregon.gov/gov/Documents/executive_orders/eo_20-07.pdfhttps://www.oregon.gov/gov/Documents/executive_orders/eo_20-12.pdf)  <https://www.edweek.org/ew/section/multimedia/map-coronavirus-and-school-closures.html>  <https://www.nytimes.com/interactive/2020/us/coronavirus-stay-at-home-order.html> |
| 38 | [https://www.governor.pa.gov/newsroom/gov-wolf-signs-covid-19-disaster-declaration-to-provide-increased-support-for-state-response/](https://www.governor.pa.gov/newsroom/gov-wolf-signs-covid-19-disaster-declaration-to-provide-increased-support-for-state-response/https://www.governor.pa.gov/newsroom/gov-wolf-puts-statewide-covid-19-mitigation-efforts-in-effect-stresses-need-for-every-pennsylvanian-to-take-action-to-stop-the-spread/https://www.governor.pa.gov/newsroom/gov-wolf-puts-statewide-covid-19-mitigation-efforts-in-effect-stresses-need-for-every-pennsylvanian-to-take-action-to-stop-the-spread/https://www.governor.pa.gov/newsroom/wolf-administration-updates-businesses-on-guidance-for-covid-19-mitigation-efforts/)  [https://www.governor.pa.gov/newsroom/gov-wolf-puts-statewide-covid-19-mitigation-efforts-in-effect-stresses-need-for-every-pennsylvanian-to-take-action-to-stop-the-spread/](https://www.governor.pa.gov/newsroom/gov-wolf-signs-covid-19-disaster-declaration-to-provide-increased-support-for-state-response/https://www.governor.pa.gov/newsroom/gov-wolf-puts-statewide-covid-19-mitigation-efforts-in-effect-stresses-need-for-every-pennsylvanian-to-take-action-to-stop-the-spread/https://www.governor.pa.gov/newsroom/gov-wolf-puts-statewide-covid-19-mitigation-efforts-in-effect-stresses-need-for-every-pennsylvanian-to-take-action-to-stop-the-spread/https://www.governor.pa.gov/newsroom/wolf-administration-updates-businesses-on-guidance-for-covid-19-mitigation-efforts/)  [https://www.governor.pa.gov/newsroom/gov-wolf-puts-statewide-covid-19-mitigation-efforts-in-effect-stresses-need-for-every-pennsylvanian-to-take-action-to-stop-the-spread/](https://www.governor.pa.gov/newsroom/gov-wolf-signs-covid-19-disaster-declaration-to-provide-increased-support-for-state-response/https://www.governor.pa.gov/newsroom/gov-wolf-puts-statewide-covid-19-mitigation-efforts-in-effect-stresses-need-for-every-pennsylvanian-to-take-action-to-stop-the-spread/https://www.governor.pa.gov/newsroom/gov-wolf-puts-statewide-covid-19-mitigation-efforts-in-effect-stresses-need-for-every-pennsylvanian-to-take-action-to-stop-the-spread/https://www.governor.pa.gov/newsroom/wolf-administration-updates-businesses-on-guidance-for-covid-19-mitigation-efforts/)  [https://www.governor.pa.gov/newsroom/wolf-administration-updates-businesses-on-guidance-for-covid-19-mitigation-efforts/](https://www.governor.pa.gov/newsroom/gov-wolf-signs-covid-19-disaster-declaration-to-provide-increased-support-for-state-response/https://www.governor.pa.gov/newsroom/gov-wolf-puts-statewide-covid-19-mitigation-efforts-in-effect-stresses-need-for-every-pennsylvanian-to-take-action-to-stop-the-spread/https://www.governor.pa.gov/newsroom/gov-wolf-puts-statewide-covid-19-mitigation-efforts-in-effect-stresses-need-for-every-pennsylvanian-to-take-action-to-stop-the-spread/https://www.governor.pa.gov/newsroom/wolf-administration-updates-businesses-on-guidance-for-covid-19-mitigation-efforts/)  <https://www.edweek.org/ew/section/multimedia/map-coronavirus-and-school-closures.html>  <https://www.nytimes.com/interactive/2020/us/coronavirus-stay-at-home-order.html>  <https://www.governor.pa.gov/newsroom/gov-wolf-sec-of-health-signs-expanded-mask-wearing-order/> |
| 39 | <https://governor.ri.gov/documents/orders/Executive-Order-20-02.pdf>  <https://governor.ri.gov/documents/orders/Executive-Order-20-04.pdf>  <https://governor.ri.gov/documents/orders/Executive-Order-20-14.pdf>  <https://governor.ri.gov/documents/orders/Executive-Order-20-04.pdf>  <https://governor.ri.gov/documents/orders/Executive-Order-20-14.pdf>  <https://www.edweek.org/ew/section/multimedia/map-coronavirus-and-school-closures.html>  <https://www.nytimes.com/interactive/2020/us/coronavirus-stay-at-home-order.html>  https://www.necn.com/news/coronavirus/with-reopening-looming-ri-gov-to-provide-update-on-coronavirus-response/2268092/ |
| 40 | [https://governor.sc.gov/news/2020-03/gov-henry-mcmaster-declare-state-emergency-order-lancaster-kershaw-county-schools](https://governor.sc.gov/news/2020-03/gov-henry-mcmaster-declare-state-emergency-order-lancaster-kershaw-county-schoolshttps://governor.sc.gov/sites/default/files/Documents/Executive-Orders/2020-03-15%20FILED%20Executive%20Order%20No.%202020-09%20-%20Closing%20Schools%20Cancelling%20Elections%20Other%20Provisions%20Due%20to%20COVID-19.pdfhttps://governor.sc.gov/sites/default/files/Documents/Executive-Orders/2020-03-17%20eFILED%20Executive%20Order%20No.%202020-10%20-%20Directing%20Additional%20Emergency%20Measures%20Due%20to%20COVID-19.pdfhttps://governor.sc.gov/sites/default/files/Documents/Executive-Orders/2020-03-23%20eFILED%20Executive%20Order%20No.%202020-13%20-%20Authorizing%20Law%20Enforcement%20to%20Preserve%20Public%20Health.pdfhttps://governor.sc.gov/sites/default/files/Documents/Executive-Orders/2020-03-17%20eFILED%20Executive%20Order%20No.%202020-10%20-%20Directing%20Additional%20Emergency%20Measures%20Due%20to%20COVID-19.pdfhttps://governor.sc.gov/sites/default/files/Documents/Executive-Orders/2020-03-31%20eFILED%20Executive%20Order%20No.%202020-17%20-%20Closure%20of%20Non-Essential%20Businesses.pdf)  [https://governor.sc.gov/sites/default/files/Documents/Executive-Orders/2020-03-15%20FILED%20Executive%20Order%20No.%202020-09%20-%20Closing%20Schools%20Cancelling%20Elections%20Other%20Provisions%20Due%20to%20COVID-19.pdf](https://governor.sc.gov/news/2020-03/gov-henry-mcmaster-declare-state-emergency-order-lancaster-kershaw-county-schoolshttps://governor.sc.gov/sites/default/files/Documents/Executive-Orders/2020-03-15%20FILED%20Executive%20Order%20No.%202020-09%20-%20Closing%20Schools%20Cancelling%20Elections%20Other%20Provisions%20Due%20to%20COVID-19.pdfhttps://governor.sc.gov/sites/default/files/Documents/Executive-Orders/2020-03-17%20eFILED%20Executive%20Order%20No.%202020-10%20-%20Directing%20Additional%20Emergency%20Measures%20Due%20to%20COVID-19.pdfhttps://governor.sc.gov/sites/default/files/Documents/Executive-Orders/2020-03-23%20eFILED%20Executive%20Order%20No.%202020-13%20-%20Authorizing%20Law%20Enforcement%20to%20Preserve%20Public%20Health.pdfhttps://governor.sc.gov/sites/default/files/Documents/Executive-Orders/2020-03-17%20eFILED%20Executive%20Order%20No.%202020-10%20-%20Directing%20Additional%20Emergency%20Measures%20Due%20to%20COVID-19.pdfhttps://governor.sc.gov/sites/default/files/Documents/Executive-Orders/2020-03-31%20eFILED%20Executive%20Order%20No.%202020-17%20-%20Closure%20of%20Non-Essential%20Businesses.pdf)  [https://governor.sc.gov/sites/default/files/Documents/Executive-Orders/2020-03-17%20eFILED%20Executive%20Order%20No.%202020-10%20-%20Directing%20Additional%20Emergency%20Measures%20Due%20to%20COVID-19.pdf](https://governor.sc.gov/news/2020-03/gov-henry-mcmaster-declare-state-emergency-order-lancaster-kershaw-county-schoolshttps://governor.sc.gov/sites/default/files/Documents/Executive-Orders/2020-03-15%20FILED%20Executive%20Order%20No.%202020-09%20-%20Closing%20Schools%20Cancelling%20Elections%20Other%20Provisions%20Due%20to%20COVID-19.pdfhttps://governor.sc.gov/sites/default/files/Documents/Executive-Orders/2020-03-17%20eFILED%20Executive%20Order%20No.%202020-10%20-%20Directing%20Additional%20Emergency%20Measures%20Due%20to%20COVID-19.pdfhttps://governor.sc.gov/sites/default/files/Documents/Executive-Orders/2020-03-23%20eFILED%20Executive%20Order%20No.%202020-13%20-%20Authorizing%20Law%20Enforcement%20to%20Preserve%20Public%20Health.pdfhttps://governor.sc.gov/sites/default/files/Documents/Executive-Orders/2020-03-17%20eFILED%20Executive%20Order%20No.%202020-10%20-%20Directing%20Additional%20Emergency%20Measures%20Due%20to%20COVID-19.pdfhttps://governor.sc.gov/sites/default/files/Documents/Executive-Orders/2020-03-31%20eFILED%20Executive%20Order%20No.%202020-17%20-%20Closure%20of%20Non-Essential%20Businesses.pdf)  [https://governor.sc.gov/sites/default/files/Documents/Executive-Orders/2020-03-23%20eFILED%20Executive%20Order%20No.%202020-13%20-%20Authorizing%20Law%20Enforcement%20to%20Preserve%20Public%20Health.pdf](https://governor.sc.gov/news/2020-03/gov-henry-mcmaster-declare-state-emergency-order-lancaster-kershaw-county-schoolshttps://governor.sc.gov/sites/default/files/Documents/Executive-Orders/2020-03-15%20FILED%20Executive%20Order%20No.%202020-09%20-%20Closing%20Schools%20Cancelling%20Elections%20Other%20Provisions%20Due%20to%20COVID-19.pdfhttps://governor.sc.gov/sites/default/files/Documents/Executive-Orders/2020-03-17%20eFILED%20Executive%20Order%20No.%202020-10%20-%20Directing%20Additional%20Emergency%20Measures%20Due%20to%20COVID-19.pdfhttps://governor.sc.gov/sites/default/files/Documents/Executive-Orders/2020-03-23%20eFILED%20Executive%20Order%20No.%202020-13%20-%20Authorizing%20Law%20Enforcement%20to%20Preserve%20Public%20Health.pdfhttps://governor.sc.gov/sites/default/files/Documents/Executive-Orders/2020-03-17%20eFILED%20Executive%20Order%20No.%202020-10%20-%20Directing%20Additional%20Emergency%20Measures%20Due%20to%20COVID-19.pdfhttps://governor.sc.gov/sites/default/files/Documents/Executive-Orders/2020-03-31%20eFILED%20Executive%20Order%20No.%202020-17%20-%20Closure%20of%20Non-Essential%20Businesses.pdf)  [https://governor.sc.gov/sites/default/files/Documents/Executive-Orders/2020-03-17%20eFILED%20Executive%20Order%20No.%202020-10%20-%20Directing%20Additional%20Emergency%20Measures%20Due%20to%20COVID-19.pdf](https://governor.sc.gov/news/2020-03/gov-henry-mcmaster-declare-state-emergency-order-lancaster-kershaw-county-schoolshttps://governor.sc.gov/sites/default/files/Documents/Executive-Orders/2020-03-15%20FILED%20Executive%20Order%20No.%202020-09%20-%20Closing%20Schools%20Cancelling%20Elections%20Other%20Provisions%20Due%20to%20COVID-19.pdfhttps://governor.sc.gov/sites/default/files/Documents/Executive-Orders/2020-03-17%20eFILED%20Executive%20Order%20No.%202020-10%20-%20Directing%20Additional%20Emergency%20Measures%20Due%20to%20COVID-19.pdfhttps://governor.sc.gov/sites/default/files/Documents/Executive-Orders/2020-03-23%20eFILED%20Executive%20Order%20No.%202020-13%20-%20Authorizing%20Law%20Enforcement%20to%20Preserve%20Public%20Health.pdfhttps://governor.sc.gov/sites/default/files/Documents/Executive-Orders/2020-03-17%20eFILED%20Executive%20Order%20No.%202020-10%20-%20Directing%20Additional%20Emergency%20Measures%20Due%20to%20COVID-19.pdfhttps://governor.sc.gov/sites/default/files/Documents/Executive-Orders/2020-03-31%20eFILED%20Executive%20Order%20No.%202020-17%20-%20Closure%20of%20Non-Essential%20Businesses.pdf)  [https://governor.sc.gov/sites/default/files/Documents/Executive-Orders/2020-03-31%20eFILED%20Executive%20Order%20No.%202020-17%20-%20Closure%20of%20Non-Essential%20Businesses.pdf](https://governor.sc.gov/news/2020-03/gov-henry-mcmaster-declare-state-emergency-order-lancaster-kershaw-county-schoolshttps://governor.sc.gov/sites/default/files/Documents/Executive-Orders/2020-03-15%20FILED%20Executive%20Order%20No.%202020-09%20-%20Closing%20Schools%20Cancelling%20Elections%20Other%20Provisions%20Due%20to%20COVID-19.pdfhttps://governor.sc.gov/sites/default/files/Documents/Executive-Orders/2020-03-17%20eFILED%20Executive%20Order%20No.%202020-10%20-%20Directing%20Additional%20Emergency%20Measures%20Due%20to%20COVID-19.pdfhttps://governor.sc.gov/sites/default/files/Documents/Executive-Orders/2020-03-23%20eFILED%20Executive%20Order%20No.%202020-13%20-%20Authorizing%20Law%20Enforcement%20to%20Preserve%20Public%20Health.pdfhttps://governor.sc.gov/sites/default/files/Documents/Executive-Orders/2020-03-17%20eFILED%20Executive%20Order%20No.%202020-10%20-%20Directing%20Additional%20Emergency%20Measures%20Due%20to%20COVID-19.pdfhttps://governor.sc.gov/sites/default/files/Documents/Executive-Orders/2020-03-31%20eFILED%20Executive%20Order%20No.%202020-17%20-%20Closure%20of%20Non-Essential%20Businesses.pdf)  <https://ballotpedia.org/Travel_restrictions_issued_by_states_in_response_to_the_coronavirus_(COVID-19)_pandemic,_2020>  <https://www.edweek.org/ew/section/multimedia/map-coronavirus-and-school-closures.html>  <https://www.nytimes.com/interactive/2020/us/coronavirus-stay-at-home-order.html> |
| 41 | [https://sdsos.gov/general-information/executive-actions/executive-orders/assets/2020-04.PDF](https://sdsos.gov/general-information/executive-actions/executive-orders/assets/2020-04.PDF%0a)  <https://www.edweek.org/ew/section/multimedia/map-coronavirus-and-school-closures.html> |
| 42 | <https://publications.tnsosfiles.com/pub/execorders/exec-orders-lee15.pdf>  <https://publications.tnsosfiles.com/pub/execorders/exec-orders-lee17.pdf>  <https://www.tn.gov/governor/covid-19/covid-19-daily-bulletin/2020/3/23/covid-19-bulletin--4---march-23--2020.html>  <https://www.tn.gov/governor/covid-19/covid-19-daily-bulletin/2020/3/30/covid-19-bulletin--8.html>  <https://www.edweek.org/ew/section/multimedia/map-coronavirus-and-school-closures.html>  <https://www.nytimes.com/interactive/2020/us/coronavirus-stay-at-home-order.html> |
| 43 | [https://gov.texas.gov/news/post/governor-abbott-holds-press-conference-on-coronavirus-declares-state-of-disaster-for-all-texas-counties](https://gov.texas.gov/news/post/governor-abbott-holds-press-conference-on-coronavirus-declares-state-of-disaster-for-all-texas-countieshttps://gov.texas.gov/news/post/governor-abbott-issues-executive-orders-in-accordance-with-federal-guidelines-to-mitigate-spread-of-covid-19-in-texashttps://gov.texas.gov/news/post/governor-abbott-issues-executive-orders-in-accordance-with-federal-guidelines-to-mitigate-spread-of-covid-19-in-texas)  [https://gov.texas.gov/news/post/governor-abbott-issues-executive-orders-in-accordance-with-federal-guidelines-to-mitigate-spread-of-covid-19-in-texas](https://gov.texas.gov/news/post/governor-abbott-holds-press-conference-on-coronavirus-declares-state-of-disaster-for-all-texas-countieshttps://gov.texas.gov/news/post/governor-abbott-issues-executive-orders-in-accordance-with-federal-guidelines-to-mitigate-spread-of-covid-19-in-texashttps://gov.texas.gov/news/post/governor-abbott-issues-executive-orders-in-accordance-with-federal-guidelines-to-mitigate-spread-of-covid-19-in-texas)  [https://gov.texas.gov/news/post/governor-abbott-issues-executive-orders-in-accordance-with-federal-guidelines-to-mitigate-spread-of-covid-19-in-texas](https://gov.texas.gov/news/post/governor-abbott-holds-press-conference-on-coronavirus-declares-state-of-disaster-for-all-texas-countieshttps://gov.texas.gov/news/post/governor-abbott-issues-executive-orders-in-accordance-with-federal-guidelines-to-mitigate-spread-of-covid-19-in-texashttps://gov.texas.gov/news/post/governor-abbott-issues-executive-orders-in-accordance-with-federal-guidelines-to-mitigate-spread-of-covid-19-in-texas)  <https://ballotpedia.org/Travel_restrictions_issued_by_states_in_response_to_the_coronavirus_(COVID-19)_pandemic,_2020>  <https://www.edweek.org/ew/section/multimedia/map-coronavirus-and-school-closures.html>  <https://www.nytimes.com/interactive/2020/us/coronavirus-stay-at-home-order.html> |
| 44 | <https://rules.utah.gov/wp-content/uploads/Utah-Executive-Order-No.-2020-1.pdf>  <https://www.edweek.org/ew/section/multimedia/map-coronavirus-and-school-closures.html>  <https://www.nytimes.com/interactive/2020/us/coronavirus-stay-at-home-order.html> |
| 45 | <https://governor.vermont.gov/press-release/governor-phil-scott-declares-state-emergency-implement-new-covid-19-community>  <https://governor.vermont.gov/press-release/governor-phil-scott-announces-new-guidance-covid-19-community-mitigation-measures>  <https://governor.vermont.gov/press-release/governor-phil-scott-announces-new-covid-19-community-mitigation-measures-directs>  <https://governor.vermont.gov/press-release/governor-phil-scott-announces-new-guidance-covid-19-community-mitigation-measures>  <https://governor.vermont.gov/press-release/governor-phil-scott-issues-%E2%80%9Cstay-home-stay-safe%E2%80%9D-order-directs-additional-closures>  <https://ballotpedia.org/Travel_restrictions_issued_by_states_in_response_to_the_coronavirus_(COVID-19)_pandemic,_2020>  <https://www.edweek.org/ew/section/multimedia/map-coronavirus-and-school-closures.html>  <https://www.nytimes.com/interactive/2020/us/coronavirus-stay-at-home-order.html> |
| 46 | <https://www.governor.virginia.gov/media/governorvirginiagov/governor-of-virginia/pdf/eo/EO-51-Declaration-of-a-State-of-Emergency-Due-to-Novel-Coronavirus-(COVID-19).pdf>  <https://www.virginia.gov/coronavirus/public-health-and-safety/#855776>  <https://www.virginia.gov/coronavirus/business-operations/#855823>  <https://www.virginia.gov/coronavirus/business-operations/#855823>  <https://www.edweek.org/ew/section/multimedia/map-coronavirus-and-school-closures.html>  <https://www.nytimes.com/interactive/2020/us/coronavirus-stay-at-home-order.html> |
| 47 | [https://www.governor.wa.gov/news-media/inslee-issues-covid-19-emergency-proclamation](https://www.governor.wa.gov/news-media/inslee-issues-covid-19-emergency-proclamationhttps://www.governor.wa.gov/news-media/inslee-issues-emergency-proclamation-limits-large-events-minimize-public-health-riskhttps://www.governor.wa.gov/news-media/inslee-statement-statewide-shutdown-restaurants-bars-and-limits-size-gatherings-expandedhttps://www.governor.wa.gov/news-media/inslee-announces-stay-home-stay-healthy%C2%A0orderhttps://www.governor.wa.gov/news-media/inslee-announces-statewide-shutdown-restaurants-bars-and-expanded-social-gathering-limits)  [https://www.governor.wa.gov/news-media/inslee-issues-emergency-proclamation-limits-large-events-minimize-public-health-risk](https://www.governor.wa.gov/news-media/inslee-issues-covid-19-emergency-proclamationhttps://www.governor.wa.gov/news-media/inslee-issues-emergency-proclamation-limits-large-events-minimize-public-health-riskhttps://www.governor.wa.gov/news-media/inslee-statement-statewide-shutdown-restaurants-bars-and-limits-size-gatherings-expandedhttps://www.governor.wa.gov/news-media/inslee-announces-stay-home-stay-healthy%C2%A0orderhttps://www.governor.wa.gov/news-media/inslee-announces-statewide-shutdown-restaurants-bars-and-expanded-social-gathering-limits)  [https://www.governor.wa.gov/news-media/inslee-statement-statewide-shutdown-restaurants-bars-and-limits-size-gatherings-expanded](https://www.governor.wa.gov/news-media/inslee-issues-covid-19-emergency-proclamationhttps://www.governor.wa.gov/news-media/inslee-issues-emergency-proclamation-limits-large-events-minimize-public-health-riskhttps://www.governor.wa.gov/news-media/inslee-statement-statewide-shutdown-restaurants-bars-and-limits-size-gatherings-expandedhttps://www.governor.wa.gov/news-media/inslee-announces-stay-home-stay-healthy%C2%A0orderhttps://www.governor.wa.gov/news-media/inslee-announces-statewide-shutdown-restaurants-bars-and-expanded-social-gathering-limits)  [https://www.governor.wa.gov/news-media/inslee-announces-stay-home-stay-healthy%C2%A0order](https://www.governor.wa.gov/news-media/inslee-issues-covid-19-emergency-proclamationhttps://www.governor.wa.gov/news-media/inslee-issues-emergency-proclamation-limits-large-events-minimize-public-health-riskhttps://www.governor.wa.gov/news-media/inslee-statement-statewide-shutdown-restaurants-bars-and-limits-size-gatherings-expandedhttps://www.governor.wa.gov/news-media/inslee-announces-stay-home-stay-healthy%C2%A0orderhttps://www.governor.wa.gov/news-media/inslee-announces-statewide-shutdown-restaurants-bars-and-expanded-social-gathering-limits)  [https://www.governor.wa.gov/news-media/inslee-announces-statewide-shutdown-restaurants-bars-and-expanded-social-gathering-limits](https://www.governor.wa.gov/news-media/inslee-issues-covid-19-emergency-proclamationhttps://www.governor.wa.gov/news-media/inslee-issues-emergency-proclamation-limits-large-events-minimize-public-health-riskhttps://www.governor.wa.gov/news-media/inslee-statement-statewide-shutdown-restaurants-bars-and-limits-size-gatherings-expandedhttps://www.governor.wa.gov/news-media/inslee-announces-stay-home-stay-healthy%C2%A0orderhttps://www.governor.wa.gov/news-media/inslee-announces-statewide-shutdown-restaurants-bars-and-expanded-social-gathering-limits)  <https://www.edweek.org/ew/section/multimedia/map-coronavirus-and-school-closures.html>  <https://www.nytimes.com/interactive/2020/us/coronavirus-stay-at-home-order.html> |
| 48 | [https://governor.wv.gov/News/press-releases/2020/Pages/COVID-19-UPDATE-Executive-Order-limiting-restaurants-and-bars,-closing-casinos-statewide.aspx](https://governor.wv.gov/News/press-releases/2020/Pages/COVID-19-UPDATE-Executive-Order-limiting-restaurants-and-bars,-closing-casinos-statewide.aspxhttps://governor.wv.gov/News/press-releases/2020/Pages/COVID-19-UPDATE-Gov.-Justice-holds-statewide-address-to-discuss-coronavirus-precautions.aspxhttps://governor.wv.gov/News/press-releases/2020/Pages/COVID-19-UPDATE-Gov.-Justice-state-officials-announce-closure-of-gyms-and-recreation-facilities.aspx)  [https://governor.wv.gov/News/press-releases/2020/Pages/COVID-19-UPDATE-Gov.-Justice-holds-statewide-address-to-discuss-coronavirus-precautions.aspx](https://governor.wv.gov/News/press-releases/2020/Pages/COVID-19-UPDATE-Executive-Order-limiting-restaurants-and-bars,-closing-casinos-statewide.aspxhttps://governor.wv.gov/News/press-releases/2020/Pages/COVID-19-UPDATE-Gov.-Justice-holds-statewide-address-to-discuss-coronavirus-precautions.aspxhttps://governor.wv.gov/News/press-releases/2020/Pages/COVID-19-UPDATE-Gov.-Justice-state-officials-announce-closure-of-gyms-and-recreation-facilities.aspx)  [https://governor.wv.gov/News/press-releases/2020/Pages/COVID-19-UPDATE-Gov.-Justice-state-officials-announce-closure-of-gyms-and-recreation-facilities.aspx](https://governor.wv.gov/News/press-releases/2020/Pages/COVID-19-UPDATE-Executive-Order-limiting-restaurants-and-bars,-closing-casinos-statewide.aspxhttps://governor.wv.gov/News/press-releases/2020/Pages/COVID-19-UPDATE-Gov.-Justice-holds-statewide-address-to-discuss-coronavirus-precautions.aspxhttps://governor.wv.gov/News/press-releases/2020/Pages/COVID-19-UPDATE-Gov.-Justice-state-officials-announce-closure-of-gyms-and-recreation-facilities.aspx)  <https://ballotpedia.org/Travel_restrictions_issued_by_states_in_response_to_the_coronavirus_(COVID-19)_pandemic,_2020>  <https://www.edweek.org/ew/section/multimedia/map-coronavirus-and-school-closures.html>  <https://www.nytimes.com/interactive/2020/us/coronavirus-stay-at-home-order.html> |
| 49 | [https://evers.wi.gov/Documents/COVID19/EMO12-SaferAtHome.pdf](https://evers.wi.gov/Documents/COVID19/EMO12-SaferAtHome.pdfhttps://content.govdelivery.com/accounts/WIGOV/bulletins/281a7bchttps://evers.wi.gov/Documents/COVID19/EMO12-SaferAtHome.pdfhttps://evers.wi.gov/Documents/COVID19/EMO12-SaferAtHome.pdfhttps://evers.wi.gov/Documents/COVID19/EMO12-SaferAtHome.pdf)  [https://content.govdelivery.com/accounts/WIGOV/bulletins/281a7bc](https://evers.wi.gov/Documents/COVID19/EMO12-SaferAtHome.pdfhttps://content.govdelivery.com/accounts/WIGOV/bulletins/281a7bchttps://evers.wi.gov/Documents/COVID19/EMO12-SaferAtHome.pdfhttps://evers.wi.gov/Documents/COVID19/EMO12-SaferAtHome.pdfhttps://evers.wi.gov/Documents/COVID19/EMO12-SaferAtHome.pdf)  [https://evers.wi.gov/Documents/COVID19/EMO12-SaferAtHome.pdf](https://evers.wi.gov/Documents/COVID19/EMO12-SaferAtHome.pdfhttps://content.govdelivery.com/accounts/WIGOV/bulletins/281a7bchttps://evers.wi.gov/Documents/COVID19/EMO12-SaferAtHome.pdfhttps://evers.wi.gov/Documents/COVID19/EMO12-SaferAtHome.pdfhttps://evers.wi.gov/Documents/COVID19/EMO12-SaferAtHome.pdf)  [https://evers.wi.gov/Documents/COVID19/EMO12-SaferAtHome.pdf](https://evers.wi.gov/Documents/COVID19/EMO12-SaferAtHome.pdfhttps://content.govdelivery.com/accounts/WIGOV/bulletins/281a7bchttps://evers.wi.gov/Documents/COVID19/EMO12-SaferAtHome.pdfhttps://evers.wi.gov/Documents/COVID19/EMO12-SaferAtHome.pdfhttps://evers.wi.gov/Documents/COVID19/EMO12-SaferAtHome.pdf)  [https://evers.wi.gov/Documents/COVID19/EMO12-SaferAtHome.pdf](https://evers.wi.gov/Documents/COVID19/EMO12-SaferAtHome.pdfhttps://content.govdelivery.com/accounts/WIGOV/bulletins/281a7bchttps://evers.wi.gov/Documents/COVID19/EMO12-SaferAtHome.pdfhttps://evers.wi.gov/Documents/COVID19/EMO12-SaferAtHome.pdfhttps://evers.wi.gov/Documents/COVID19/EMO12-SaferAtHome.pdf)  <https://www.edweek.org/ew/section/multimedia/map-coronavirus-and-school-closures.html>  <https://www.nytimes.com/interactive/2020/us/coronavirus-stay-at-home-order.html> |
| 50 | <https://covid19.wyo.gov/governors-orders>  <https://trib.com/news/local/casper/businesses-worry-after-governor-orders-closures-of-bars-restaurants/article_902660e7-88a3-5f8e-a267-7802f785aa12.html>  <https://www.edweek.org/ew/section/multimedia/map-coronavirus-and-school-closures.html> |

**Supplementary Table 3. Dates of symptom onset of infector-infectee pairs**

| **No.** | **Country** | **Infector.onset.lowbound** | **Infector.onset.upbound** | **Infectee.onset** |
| --- | --- | --- | --- | --- |
| 1 | China | 1/1/2020 | 1/4/2020 | 1/8/2020 |
| 2 | China | 1/10/2020 | 1/10/2020 | 1/15/2020 |
| 3 | China | 1/10/2020 | 1/10/2020 | 1/17/2020 |
| 4 | China | 1/12/2020 | 1/12/2020 | 1/25/2020 |
| 5 | China | 1/12/2020 | 1/15/2020 | 1/22/2020 |
| 6 | China | 1/14/2020 | 1/15/2020 | 1/24/2020 |
| 7 | China | 1/15/2020 | 1/15/2020 | 1/24/2020 |
| 8 | China | 1/15/2020 | 1/18/2020 | 1/18/2020 |
| 9 | China | 1/16/2020 | 1/16/2020 | 1/25/2020 |
| 10 | China | 1/17/2020 | 1/17/2020 | 1/31/2020 |
| 11 | China | 1/17/2020 | 1/17/2020 | 1/28/2020 |
| 12 | Vietnam | 1/17/2020 | 1/17/2020 | 1/20/2020 |
| 13 | China | 1/18/2020 | 1/18/2020 | 1/22/2020 |
| 14 | China | 1/18/2020 | 1/18/2020 | 1/22/2020 |
| 15 | China | 1/18/2020 | 1/18/2020 | 1/25/2020 |
| 16 | China | 1/18/2020 | 1/18/2020 | 1/24/2020 |
| 17 | China | 1/18/2020 | 1/18/2020 | 1/25/2020 |
| 18 | China | 1/19/2020 | 1/19/2020 | 2/4/2020 |
| 19 | China | 1/20/2020 | 1/20/2020 | 1/24/2020 |
| 20 | China | 1/20/2020 | 1/20/2020 | 1/22/2020 |
| 21 | China | 1/20/2020 | 1/20/2020 | 1/23/2020 |
| 22 | China | 1/20/2020 | 1/20/2020 | 1/25/2020 |
| 23 | China | 1/20/2020 | 1/20/2020 | 1/26/2020 |
| 24 | China | 1/20/2020 | 1/20/2020 | 2/6/2020 |
| 25 | South Korea | 1/20/2020 | 1/20/2020 | 1/29/2020 |
| 26 | Germany | 1/21/2020 | 1/21/2020 | 1/24/2020 |
| 27 | Germany | 1/21/2020 | 1/21/2020 | 1/24/2020 |
| 28 | China | 1/21/2020 | 1/21/2020 | 2/2/2020 |
| 29 | China | 1/21/2020 | 1/21/2020 | 1/27/2020 |
| 30 | China | 1/22/2020 | 1/22/2020 | 1/24/2020 |
| 31 | China | 1/22/2020 | 1/22/2020 | 1/30/2020 |
| 32 | China | 1/22/2020 | 1/22/2020 | 1/19/2020 |
| 33 | China | 1/22/2020 | 1/22/2020 | 1/28/2020 |
| 34 | South Korea | 1/22/2020 | 1/22/2020 | 1/26/2020 |
| 35 | United States | 1/22/2020 | 1/23/2020 | 1/28/2020 |
| 36 | China | 1/22/2020 | 1/25/2020 | 1/28/2020 |
| 37 | China | 1/22/2020 | 1/30/2020 | 2/4/2020 |
| 38 | China | 1/22/2020 | 1/22/2020 | 1/28/2020 |
| 39 | China | 1/22/2020 | 1/22/2020 | 1/25/2020 |
| 40 | China | 1/22/2020 | 1/22/2020 | 1/24/2020 |
| 41 | China | 1/22/2020 | 1/22/2020 | 1/22/2020 |
| 42 | China | 1/22/2020 | 1/22/2020 | 1/24/2020 |
| 43 | China | 1/23/2020 | 1/23/2020 | 2/3/2020 |
| 44 | China | 1/21/2020 | 1/23/2020 | 1/27/2020 |
| 45 | China | 1/21/2020 | 1/23/2020 | 1/27/2020 |
| 46 | Germany | 1/24/2020 | 1/24/2020 | 1/26/2020 |
| 47 | Germany | 1/24/2020 | 1/24/2020 | 1/26/2020 |
| 48 | China | 1/24/2020 | 1/24/2020 | 2/1/2020 |
| 49 | China | 1/24/2020 | 1/24/2020 | 1/25/2020 |
| 50 | China | 1/24/2020 | 1/24/2020 | 1/31/2020 |
| 51 | China | 1/24/2020 | 1/24/2020 | 2/1/2020 |
| 52 | China | 1/24/2020 | 1/24/2020 | 1/26/2020 |
| 53 | China | 1/24/2020 | 1/24/2020 | 1/26/2020 |
| 54 | China | 1/24/2020 | 1/24/2020 | 1/25/2020 |
| 55 | China | 1/24/2020 | 1/26/2020 | 2/5/2020 |
| 56 | China | 1/24/2020 | 1/26/2020 | 2/5/2020 |
| 57 | China | 1/25/2020 | 1/25/2020 | 1/28/2020 |
| 58 | China | 1/25/2020 | 1/25/2020 | 1/27/2020 |
| 59 | China | 1/25/2020 | 1/25/2020 | 2/3/2020 |
| 60 | China | 1/25/2020 | 1/25/2020 | 1/26/2020 |
| 61 | Vietnam | 1/25/2020 | 1/25/2020 | 1/31/2020 |
| 62 | Vietnam | 1/25/2020 | 1/25/2020 | 2/3/2020 |
| 63 | Vietnam | 1/25/2020 | 1/25/2020 | 2/3/2020 |
| 64 | China | 1/25/2020 | 1/28/2020 | 1/31/2020 |
| 65 | China | 1/25/2020 | 1/31/2020 | 1/28/2020 |
| 66 | China | 1/25/2020 | 1/31/2020 | 1/26/2020 |
| 67 | China | 1/25/2020 | 1/31/2020 | 1/25/2020 |
| 68 | China | 1/25/2020 | 1/31/2020 | 1/25/2020 |
| 69 | China | 1/25/2020 | 1/31/2020 | 1/28/2020 |
| 70 | China | 1/25/2020 | 1/31/2020 | 1/27/2020 |
| 71 | China | 1/26/2020 | 1/26/2020 | 1/30/2020 |
| 72 | China | 1/26/2020 | 1/26/2020 | 2/5/2020 |
| 73 | South Korea | 1/26/2020 | 1/26/2020 | 1/30/2020 |
| 74 | South Korea | 1/26/2020 | 1/26/2020 | 1/29/2020 |
| 75 | South Korea | 1/26/2020 | 1/26/2020 | 1/30/2020 |
| 76 | South Korea | 1/26/2020 | 1/26/2020 | 1/30/2020 |
| 77 | China | 1/26/2020 | 1/26/2020 | 1/26/2020 |
| 78 | China | 1/10/2020 | 1/29/2020 | 1/22/2020 |
| 79 | China | 1/23/2020 | 1/26/2020 | 1/30/2020 |
| 80 | China | 1/26/2020 | 1/26/2020 | 2/2/2020 |
| 81 | China | 1/27/2020 | 1/27/2020 | 1/31/2020 |
| 82 | China | 1/27/2020 | 1/28/2020 | 2/1/2020 |
| 83 | China | 1/27/2020 | 1/28/2020 | 1/28/2020 |
| 84 | China | 1/27/2020 | 1/28/2020 | 1/31/2020 |
| 85 | China | 1/28/2020 | 1/28/2020 | 1/24/2020 |
| 86 | Singapore | 1/28/2020 | 1/28/2020 | 1/31/2020 |
| 87 | China | 1/28/2020 | 2/3/2020 | 2/8/2020 |
| 88 | China | 1/29/2020 | 1/29/2020 | 2/1/2020 |
| 89 | China | 1/29/2020 | 1/29/2020 | 2/1/2020 |
| 90 | Malaysia | 1/29/2020 | 1/29/2020 | 2/1/2020 |
| 91 | Malaysia | 1/29/2020 | 1/29/2020 | 2/1/2020 |
| 92 | Malaysia | 1/29/2020 | 1/29/2020 | 2/5/2020 |
| 93 | China | 1/29/2020 | 1/29/2020 | 2/1/2020 |
| 94 | China | 1/29/2020 | 1/29/2020 | 2/6/2020 |
| 95 | China | 1/29/2020 | 1/29/2020 | 2/3/2020 |
| 96 | China | 1/29/2020 | 1/29/2020 | 2/3/2020 |
| 97 | China | 1/29/2020 | 1/29/2020 | 2/5/2020 |
| 98 | Singapore | 1/29/2020 | 1/29/2020 | 2/1/2020 |
| 99 | Singapore | 1/29/2020 | 1/29/2020 | 2/2/2020 |
| 100 | China | 1/30/2020 | 1/30/2020 | 2/3/2020 |
| 101 | China | 1/30/2020 | 1/30/2020 | 2/2/2020 |
| 102 | China | 1/30/2020 | 1/30/2020 | 2/1/2020 |
| 103 | Vietnam | 1/31/2020 | 1/31/2020 | 2/6/2020 |
| 104 | China | 1/4/2020 | 1/4/2020 | 1/11/2020 |
| 105 | China | 1/4/2020 | 1/4/2020 | 1/12/2020 |
| 106 | China | 1/4/2020 | 1/12/2020 | 1/25/2020 |
| 107 | China | 12/12/2019 | 12/12/2019 | 12/19/2019 |
| 108 | China | 12/20/2019 | 12/20/2019 | 12/25/2019 |
| 109 | China | 12/20/2019 | 12/20/2019 | 12/29/2019 |
| 110 | China | 12/21/2019 | 12/21/2019 | 12/24/2019 |
| 111 | China | 12/27/2019 | 12/27/2019 | 1/3/2020 |
| 112 | South Korea | 2/1/2020 | 2/1/2020 | 2/5/2020 |
| 113 | China | 2/12/2020 | 2/12/2020 | 2/12/2020 |
| 114 | China | 2/12/2020 | 2/12/2020 | 2/16/2020 |
| 115 | China | 2/13/2020 | 2/13/2020 | 2/25/2020 |
| 116 | China | 2/13/2020 | 2/13/2020 | 2/29/2020 |
| 117 | Japan | 2/13/2020 | 2/13/2020 | 2/15/2020 |
| 118 | Japan | 2/13/2020 | 2/13/2020 | 2/25/2020 |
| 119 | Japan | 2/13/2020 | 2/13/2020 | 2/17/2020 |
| 120 | Japan | 2/15/2020 | 2/15/2020 | 2/17/2020 |
| 121 | Japan | 2/15/2020 | 2/15/2020 | 2/17/2020 |
| 122 | Japan | 2/17/2020 | 2/17/2020 | 2/19/2020 |
| 123 | Japan | 2/17/2020 | 2/17/2020 | 2/19/2020 |
| 124 | Japan | 2/20/2020 | 2/20/2020 | 2/20/2020 |
| 125 | Japan | 2/20/2020 | 2/20/2020 | 3/3/2020 |
| 126 | Japan | 2/24/2020 | 2/24/2020 | 2/29/2020 |
| 127 | Japan | 2/29/2020 | 2/29/2020 | 3/4/2020 |
| 128 | Japan | 2/3/2020 | 2/3/2020 | 2/17/2020 |
| 129 | China | 2/6/2020 | 2/6/2020 | 2/24/2020 |
| 130 | China | 2/9/2020 | 2/9/2020 | 2/12/2020 |
| 131 | China | 2/9/2020 | 2/9/2020 | 2/10/2020 |
| 132 | China | 2/9/2020 | 2/9/2020 | 2/10/2020 |
| 133 | Japan | 2/3/2020 | 2/9/2020 | 2/24/2020 |

**Supplementary Table 4. Risk Ratios (RR) of each variable to the response variable** $\boldsymbol{R}_{\boldsymbol{t}}$

| **Independent variables** | **RR^a^ (95% CI)** | **RR^b^ (95% CI)** |
| --- | --- | --- |
| Stay-at-home | 0.8(0.76,0.84) | 0.49(0.43,0.54) |
| Face Mask | 0.71(0.58,0.85) | 0.71(0.58,0.85) |
| Gathering Ban(more than 10) | 0.86(0.83,0.9) | 0.81(0.76,0.86) |
| Non-essential Business Closure | 0.93(0.89,0.97) | 0.84(0.79,0.9) |
| Declaration of Emergency | 0.87(0.83,0.92) | 0.87(0.83,0.92) |
| Interstate Travel Restriction | 0.89(0.84,0.95) | 0.89(0.84,0.95) |
| School Closure | 0.9(0.86,0.93) | 0.9(0.86,0.93) |
| Initial Business Closure | 0.9(0.86,0.94) | 0.9(0.86,0.94) |
| Gathering Ban (more than 50) | 0.93(0.89,0.98) | 0.93(0.89,0.98) |
| Population Density | 1.25(1.19,1.33) | 1.25(1.19,1.33) |

a, the raw risk ratios from the GLM model; b, the actual risk ratios recalculated based on the inclusion relation of some interventions.

**Supplementary Table 5. Risk Ratios (RR) of each variable to the response variable** $\boldsymbol{R}_{\boldsymbol{t}}$ **(Shenzhen)**

| **Independent variables** | **RR^a^ (95% CI)** | **RR^b^ (95% CI)** |
| --- | --- | --- |
| Stay-at-home | 0.83(0.79,0.88) | 0.54(0.49,0.6) |
| Face Mask | 0.73(0.6,0.87) | 0.73(0.6,0.87) |
| Gathering Ban(more than 10) | 0.88(0.84,0.91) | 0.83(0.79,0.87) |
| Declaration of Emergency | 0.84(0.8,0.89) | 0.84(0.8,0.89) |
| Non-essential Business Closure | 0.96(0.92,1) | 0.88(0.83,0.93) |
| School Closure | 0.89(0.86,0.92) | 0.89(0.86,0.92) |
| Interstate Travel Restriction | 0.9(0.85,0.96) | 0.9(0.85,0.96) |
| Initial Business Closure | 0.92(0.88,0.96) | 0.92(0.88,0.96) |
| Gathering Ban (more than 50) | 0.95(0.91,0.99) | 0.95(0.91,0.99) |
| Population Density | 1.23(1.18,1.3) | 1.23(1.18,1.3) |

a, the raw risk ratios from the GLM model; b, the actual risk ratios recalculated based on the inclusion relation of some interventions.

**Supplementary Table 6. Risk Ratios (RR) of each variable to the response variable** $\boldsymbol{R}_{\boldsymbol{t}}$ **(Wenzhou)**

| **Independent variables** | **RR^a^ (95% CI)** | **RR^b^ (95% CI)** |
| --- | --- | --- |
| Stay-at-home | 0.82(0.78,0.86) | 0.54(0.48,0.59) |
| Face Mask | 0.72(0.56,0.85) | 0.72(0.56,0.85) |
| Gathering Ban(more than 10) | 0.88(0.84,0.91) | 0.83(0.79,0.87) |
| Declaration of Emergency | 0.87(0.83,0.9) | 0.87(0.83,0.9) |
| Non-essential Business Closure | 0.95(0.91,0.99) | 0.87(0.82,0.92) |
| Interstate Travel Restriction | 0.9(0.85,0.96) | 0.9(0.85,0.96) |
| School Closure | 0.9(0.87,0.93) | 0.9(0.87,0.93) |
| Initial Business Closure | 0.91(0.88,0.95) | 0.91(0.88,0.95) |
| Gathering Ban (more than 50) | 0.95(0.91,0.98) | 0.95(0.91,0.98) |
| Population Density | 1.22(1.17,1.28) | 1.22(1.17,1.28) |

a, the raw risk ratios from the GLM model; b, the actual risk ratios recalculated based on the inclusion relation of some interventions.
